# Meta-analysis of transcriptomic responses as a means to identify pulmonary disease outcomes for engineered nanomaterials

**DOI:** 10.1186/s12989-016-0137-5

**Published:** 2016-05-11

**Authors:** Jake Nikota, Andrew Williams, Carole L. Yauk, Håkan Wallin, Ulla Vogel, Sabina Halappanavar

**Affiliations:** 1 Environmental Health Science and Research Bureau, Health Canada, Ottawa, ON K1A 0K9 Canada; 2 National Research Centre for the Working Environment, Lerso Parkallé 105, Copenhagen, DK-2100 Denmark; 3 Department of Public Health, University of Copenhagen, Copenhagen K, DK-1353 Denmark; 4 Department of Micro- and Nanotechnology, Technical University of Denmark, DK-2800 Kgs., Lyngby, Denmark

**Keywords:** Nanomaterials, Toxicogenomics, Carbon nanotubes, Carbon black, TiO_2_ nanoparticles, Lung disease, Lung fibrosis

## Abstract

**Background:**

The increasing use of engineered nanomaterials (ENMs) of varying physical and chemical characteristics poses a great challenge for screening and assessing the potential pathology induced by these materials, necessitating novel toxicological approaches. Toxicogenomics measures changes in mRNA levels in cells and tissues following exposure to toxic substances. The resulting information on altered gene expression profiles, associated pathways, and the doses at which these changes occur, are used to identify the underlying mechanisms of toxicity and to predict disease outcomes. We evaluated the applicability of toxicogenomics data in identifying potential lung-specific (genomic datasets are currently available from experiments where mice have been exposed to various ENMs through this common route of exposure) disease outcomes following exposure to ENMs.

**Methods:**

Seven toxicogenomics studies describing mouse pulmonary responses over time following intra-tracheal exposure to increasing doses of carbon nanotubes (CNTs), carbon black, and titanium dioxide (TiO_2_) nanoparticles of varying properties were examined to understand underlying mechanisms of toxicity. mRNA profiles from these studies were compared to the publicly available datasets of 15 other mouse models of lung injury/diseases induced by various agents including bleomycin, ovalbumin, TNFα, lipopolysaccharide, bacterial infection, and welding fumes to delineate the implications of ENM-perturbed biological processes to disease pathogenesis in lungs.

**Results:**

The meta-analysis revealed two distinct clusters—one driven by TiO_2_ and the other by CNTs. Unsupervised clustering of the genes showing significant expression changes revealed that CNT response clustered with bleomycin injury and bacterial infection models, both of which are known to induce lung fibrosis, in a post-exposure-time dependent manner, irrespective of the CNT’s physical-chemical properties. TiO_2_ samples clustered separately from CNTs and disease models.

**Conclusions:**

These results indicate that in the absence of apical toxicity data, a tiered strategy beginning with short term, *in vivo* tissue transcriptomics profiling can effectively and efficiently screen new ENMs that have a higher probability of inducing pulmonary pathogenesis.

**Electronic supplementary material:**

The online version of this article (doi:10.1186/s12989-016-0137-5) contains supplementary material, which is available to authorized users.

## Background

Commercial applications of engineered nanomaterials (ENMs) continue to grow in the biomedical and manufacturing fields, resulting in an increased demand for synthesis of raw ENMs, increased potential for their release into the environment, and enhanced risk of human exposure [1].

Within the context of their nano size (1–100 nm), ENMs can be synthesized in different shapes, and with different surface charges and functionalization, all of which are shown to influence ENM-induced toxicity [2]. While there is now evidence showing that pulmonary exposure to ENMs is more hazardous than pulmonary exposure to larger particles of the same chemical composition [3–5], the implications of the observed response to adverse outcomes of regulatory importance are not understood. In addition to the inability to extrapolate toxicity data from bulk materials to ENMs, it is also unclear as to which extent toxicity data measured for a select set of ENMs can be generalized to other forms of ENMs (i.e., how to group and rank ENMs with different physicochemical characteristics). Furthermore, critical toxicological data concerning ENM exposure, dose metrics, cellular uptake, cellular fate and transport are missing. As a consequence, accurate predictions about the adverse effects of ENM exposure are not possible and a regulatory framework for human health risk assessment of these materials has yet to be established. Given the scale at which the field of nanotechnology is expanding, and the immediate need to employ conventional toxicological approaches that are time and cost intensive, new strategies are needed to complete the safety assessment of currently available ENMs in a timely manner [6].

Toxicogenomics investigates global responses induced by toxic substances at the genome, transcriptome, proteome, and metabolome levels. Toxicogenomics has been used to evaluate the potential hazards of ENMs and to identify specific ENM properties that are responsible for their toxicity. For example, we recently employed gene expression profiling to demonstrate that nano-sized titanium dioxide (nano-TiO_2_) particles of varying sizes and surface properties induce pulmonary inflammation via the same mechanisms-of-action; however, the study showed that different nano-TiO_2_ particles vary in the magnitude of the inflammatory response induced in a property-dependent manner [7]. Similarly, Poulsen et al demonstrated that pulmonary transcriptional responses indicative of lung fibrosis varied for two carbon nanotubes (CNTs) of different length, width, and content of metal impurities [8]. Although informative, these analyses produced long lists of thousands of genes, proteins, and metabolites that were identified as differentially expressed in exposed versus control tissues, making it difficult to distinguish between genes that play a role in the observed biological response from those that represent spurious events. A greater challenge lies in the identification and discrimination of genes that define benign tissue defense mechanisms from those potentially important for the progression of adverse outcomes and diseases, as many disease-causing genes are often involved in host defense. In the case of ENMs, the identification of essential transcriptional signatures of diseases or pathogenesis early after exposure will not only provide insight into the underlying mechanisms of toxicities, but will also aid in developing rapid screening tests for prioritizing ENMs for further testing, identify disease producing ENMs, and enable the identification of markers of exposure and/or effect that can be potentially used for human biomonitoring and surveillance.

Nanotoxicology research has thus far not revealed toxicities or pathologies unique to ENMs, suggesting that the adverse outcomes of exposure to ENMs can be anticipated based on existing knowledge of the toxicity or pathology induced by analogous particles, chemicals, or substances [9]. Given the number of toxicogenomics studies that have investigated toxicant-induced lung diseases, in the present study we sought to define the critical gene expression fingerprints of lung events and diseases that are overrepresented in the lung transcriptional responses induced by ENMs. We conducted a meta-analysis of previously published gene expression microarray data from mouse lungs exposed to multi-walled CNTs (MWCNTs), carbon black (CB), and nano-TiO_2_ of varying properties, and of publicly available gene expression microarray data of lung diseases induced by various exogenous agents including bleomycin, ovalbumin, TNFα, LPS, bacterial infection and welding fumes. Meta-analysis of the intersections of transcriptional changes induced by ENM and lung disease datasets were examined to identify signatures or features that are associated with ensuing lung pathology.

The study is a proof-of-principle and demonstrates the potential application of toxicogenomics data in identifying disease causing ENMs. By using publicly available lung injury models and ENMs that are known to induce lung events, we demonstrate how data derived from toxicogenomics as a stand-alone technique can inform potential disease potencies of ENMs that have not yet been extensively studied using conventional apical endpoints. The analysis was restricted to mouse models to minimize the loss of relevant biological information during the statistical normalization of cross-species data. The study analysis was also restricted to only those datasets that are readily available for download from public repositories and consisted of more than one dose in addition to controls.

## Methods

### Study selection

Table 1 summarizes the studies that were included in this analysis [7, 8, 10–19], including experimental details such as the nanomaterial/chemical, route of exposure, strain of mouse, dose, post-exposure time points investigated and microarray platform used. Since inhalation is an important route of exposure for ENMs in occupational settings, and since the pulmonary transcriptomic responses following exposure to some ENMs are fairly well characterized, studies that investigated the gene expression profiles of pulmonary responses to ENMs and lung disease models were used in the meta-analysis. The physico-chemical properties of the ENMs are summarized in Table 2. Data were obtained from the Gene Expression Omnibus (GEO) (http://www.ncbi.nlm.nih.gov/geo). The ultimate goal of this meta-analysis was to investigate the associations between gene expression profiles of lung tissue exposed to ENMs and of lung tissue from models of known pulmonary diseases.

**Table 1:** A list of the studies that were included in the meta-analysis and the experimental details

Reference	Nanomaterial/Chemical	Route of exposure	Mouse strain	Dose	Post-exposure time point	Array platform	GSE
Poulsen et al*.* (2015) [8]	Carbon nanotubes	Intratracheal	Adult C57BL/6	Single dose of 18, 54, 162 μg/mouse	1, 3, and 28 days	Agilent whole genome	GSE35284
Bourdon et al*.* (2012) [10]	Carbon black	Intratracheal	Adult C57BL/6	Single dose of 18, 54, 162 μg/mouse	1, 3, and 28 days	Agilent whole genome	GSE35193
Halappanavar et al*.* (2011) [13]	Nano titanium dioxide	Inhalation	Adult C57BL/6	1 h daily for 11 consecutive days to 42.4 mg	5 days	Agilent whole genome	GSE19487
Guo et al*.* (2012) [14]	Carbon nanotubes	Pharyngeal aspirations	Adult C57BL/6	Single dose of 0, 10, 20, 40, or 80 μg of CNT/mouse	1, 7, 28, and 56 days	Agilent whole genome	GSE29042
Reference not available	Bleomycin	Intratracheal	Adult Balb-c and C57BL6	Single dose of 0 and 1.5 U/kg body weight	24 h, 72 h, 10 day and 14 days	Affymetrix murine genome	GSE485
Erdely et al*.* (2012) [15]	Gas metal arc - stainless steel welding fume	Inhalation	Adult C57BL6	0 or 40 mg/m^3^, 3 h daily for 10 consecutive days	4 h, 14 days, 28 days	Illumina mouse bead array	GSE34056
Zeidler-Erderly et al*.* (2010) [16]	Gas metal arc—stainless steel welding fume	Pharyngeal aspiration	Adult A/J or C57BL/6 J	Four doses of 85 mg/kg	4 and 16 weeks	Illumina mouse bead array	GSE20174
Lewis et al*.* (2008) [17]	12 different lung disease model datasets including bleomycin, Th2 responses, and infection models	Differs with the model of lung disease	Differs with the model of lung disease	Differs with the model of lung disease	Differs with the model of lung disease	UCSF &Mm mouse oligo array	GSE4231
Halappanavar et al*.* (2015) [7]	Nano titanium dioxide	Intratracheal	Adult C57BL/6	Single dose of 18, 54, 162, or 486 μg/mouse	1 and 28 days	Agilent whole genome	GSE60801
Husain et al*.* (2015) [19]	Carbon Black	Intratracheal	Adult C57BL/6	Single dose of 162 μg/mouse	3 h, and 2, 3, 4, 5, 14, and 42 days	Agilent whole genome	GSE68036
Rahman et al*.* (manuscript in preparation)	Carbon nanotubes	Intratracheal	Adult C57BL/6	4 doses of 128 or 47.5 μg/mouse per week	60 days	Agilent whole genome	GSE65623
Thomson et al*.* (2012) [18]	Lung-specific TNFα over-expression	N/A	Adult C57BL/6 and transgenic mice	N/A	N/A	Agilent whole genome	GSE11037

**Table 2:** The physico-chemical properties of the ENMs investigated in the studies listed in Table 1

Reference	GSE	Name in study	Producer (particle name)	Size	BET (m^2^/g)
Poulsen et al. (2015) [8]	GSE35284	CNT_small_	Nanocyl (NC-7000)	0.85 ± 0.457 μm × 11 ± 4.5 nm	245.8
		CNT_large_	IO-LE TECNanomaterials (CP-0006-SG)	4.05 ± 2.40 μm × 67 ± 2.40 nm	14.6
Bourdon et al. (2012) [10]	GSE35193	CBNPs	Evonik/Degussa (Printex 90)	14 nm	295–338
Halappanavar et al. (2011) [13]	GSE19487	NanoTiO_2_	Kemira (UV-titan L181)	20 nm	107.7
Guo et al. (2012) [14]	GSE29042	MWCNT	Mitsui & Company (MWCNT-7)	3.86 μm × 49 ± 13.4 nm	not listed in study
Halappanavar et al. (2015) [7]	GSE60801	TiO_2_NP^10.5^	NanoAmor (NRCWE-030)	10.5 nm	139.1
		TiO_2_NP^38^	Nabond (NRCWE-025)	38 nm	28.2
		TiO_2_NP^10^	NanoAmor (NRCWE-001)	10 nm	99
		TiO_2_NP^10+^	NanoAmor (NRCWE-002)	10 nm	84
		TiO_2_NP^20.6^	Kemira (UV-Titan L181)	20.6 nm	107.7
Husain et al. (2015) [19]	GSE68036	CBNPs	Evonik/Degussa (Printex 90)	14 nm	295–338
Rahman et al. (manuscript in preparation)	GSE65623	Mitsui XNRi-7	Mitsui & Company (NRCWE-006)	5.7 ± 0.49 μm × 74 (29–173) nm	26
		CNT 401	OECD WPMNM (NM-401)	4.0 ± 0.37 μm × 67 (24–138) nm	18

### Data processing and normalization

Due to differences in microarray platforms used across the chosen studies, all gene expression data were normalized using a common statistical method. The log2 transformation was applied to all signal intensity measurements. For two color microarray studies, the LOWESS normalization method [20], using the R statistical software environment [21] was applied. For studies using Affymetrix GeneChips®, the RMA normalization was applied using the justRMA function in the affy R package [22]. Quantile normalization was applied for studies using Illumina beadchip. This was done using the lumiN function in the lumi R package [23].

Probes with technical replicates were then averaged using the median. The data for each study were then merged to their appropriate annotation files to obtain gene symbols. Probes with the same gene symbol were then averaged using the median. Experimental conditions with biological replicates were averaged using the median. The data were further normalized by subtracting the median average of the appropriate control samples resulting in the log2 fold change for each experimental condition. The control samples were then removed from the data set. All studies were then merged together using the gene symbol for data analysis.

A total of 2334 gene symbols were in common across all platforms with 190 experimental conditions relative to controls. The resulting data matrix consisting of gene symbols were filtered based on the number of experimental conditions exceeding a log2 (1.5 fold) cut-off. Although the use of fold change ranking in conjunction with a flexible (non-stringent) p-value threshold is suggested in order to generate reproducible differentially expressed gene lists [24], no numeric values as fold change or p-value cut-offs have been specifically prescribed. Using a pre-determined stringent cut-off for fold change to identify differentially expressed genes, especially in an experiment where the objective is to determine the effects following exposures to ENMs of varying potency and across several doses (including low doses where not many changes are expected), may compromise sensitivity and miss some biologically relevant genes that have low fold changes. For this reason, we have used the fold change of 1.5. Furthermore, the filtering helped remove non-informative genes that could distort correlations between experimental conditions. Gene symbols that had more than 5 experimental conditions that exceeded this cut-off (in absolute value) were retained for hierarchical cluster analysis, which resulted in 945 gene symbols.

### Dendrogram analysis

Hierarchical clustering is a statistical technique that groups experimental conditions based on distances. Hierarchical cluster analysis was conducted on normalized expression levels of the 945 gene symbols identified above. Here, the 1-Pearson correlation dissimilarity metric was used. The average linkage function, which determines how distances between sets of observations are calculated, was used and the analysis was conducted using the R software package.

### Gene Ontology Analysis

A shortlist of differentially expressed genes (DEGs) that showed differential expression in at least 50 % of the experimental conditions within the respective cluster was used in the analysis, so that only genes that are involved in the majority of experimental conditions were examined. To determine the biological relevance of the DEGs identified in the disease clusters, an analysis of the gene ontologies (GOs) enriched within the shortlisted genes of each cluster was performed. This analysis used the gene ontology consortium database, and was performed using the Cytoscape (Cytoscape Consortium) software app ClueGo (Laboratory of Integrative Cancer Immunology, Paris, France) [25, 26]. The enrichment analysis used gene associations based only on experimental evidence. GOs were included if they had a minimum of 3 genes expressed and if a minimum of 4 % of the GO was represented. To reduce the redundant GOs, GoFusion was enabled in the ClueGo software, and GOs were collapsed back to parent ontologies if all of the DEGs from the Parent and child GOs were the same. For ball and stick diagrams, a 50 % overlap of DEGs was used as the threshold for grouping of the GOs.

### Upstream Regulator analysis

Ingenuity Pathway Analysis (IPA) software (Qiagen Silicon Valley) was used to determine the upstream regulators of gene expression that might contribute to the gene expression profiles identified in the disease clusters. Specifically, the upstream regulator analysis tool was used, and only gene products, miRNA, and proteins were selected. Only upstream regulators with z-scores of 3 or more and with p < 0.001 were included.

### Fibrosis associated genes

The gene list *fibrosis of the lung* from the IPA archives was used to identify genes associated with pulmonary fibrosis. Tables and heat maps were constructed using this list as a reference.

## Results

### Comparison of transcriptomic datasets of ENM exposure versus models of lung disease

**Fig. 1:**
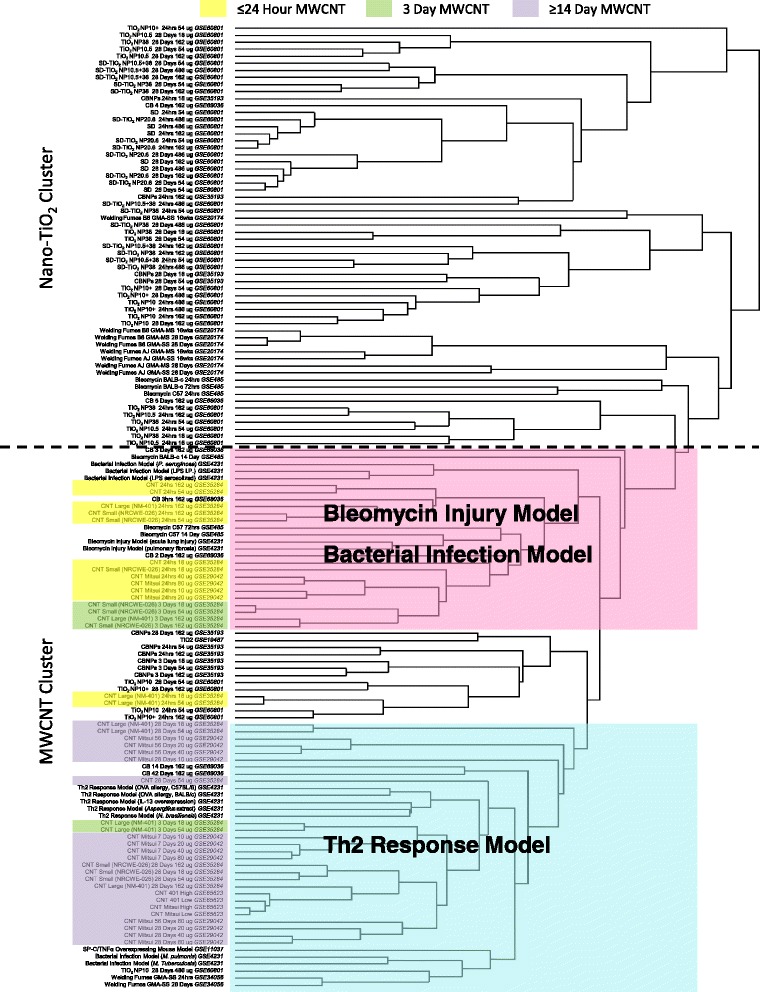
*NanoTiO*_*2*_ *and MWCNTs clustered separately, and MWCNTs were further differentiated by the post-exposure time period.* Hierarchical clustering was used to visualize the differential expression of 2334 common genes across the microarray platforms used in this study and across 137 different experimental conditions from 12 studies. The figure depicts the clustering of individual arrays performed on RNA isolated from murine lung tissue within each respective study. Two distinct clusters were identified, with nanoTiO_2_ arrays and MWCNT arrays clustering separately. The MWCNT cluster could be further divided into two sub-clusters: (1) arrays from MWCNT exposure models were correlated with those from bacteria and bleomycin exposure models (*pink*); and (2) expression profiles from MWCNT exposure models were correlated with arrays from a Th2 model (*blue*). Other nanomaterial exposure models did not cluster in notable patterns

To understand if gene expression signatures obtained from the lungs of mice exposed to nano TiO_2_, CB or MWCNTs may be associated with specific lung diseases, a meta-analysis was performed that included 12 studies that investigated lung injury or lung diseases and ENM-induced pulmonary responses (Table 1). A final data set consisted of ~700 individual microarray hybridizations representing 137 experimental conditions. Due to differences in microarray platforms used to collect the gene expression data included in this meta-analysis, only those genes that were consistently found on all of the platforms were used to derive a list of 2334 DEGs (Additional file 1: Table S1). A gene showing expression changes >1.5 relative to matched controls in more than 5 of the 137 total experimental conditions was considered to be significant and was included in the meta-analysis. Through this criterion, a total of 954 DEGs were derived. Hierarchical clustering of the 954 genes was conducted to visualize the relationship between the different experimental datasets (Fig. 1).

The key findings of this analysis, which are summarized in Fig. 1, reveal the following: 1) there were transcriptional features distinct to the response induced by bacterial infection, bleomycin induced lung injury, T helper type 2 lymphocytes (Th2)-mediated allergic response and ENMs; 2) all experimental samples exposed to nanoTiO_2_ clustered separately (Fig. 1, nanoTiO_2_ cluster) from the MWCNT-exposed samples (Fig. 1, MWCNT cluster); and, 3) only MWCNT datasets clustered with lung disease models (Fig. 1, MWCNT cluster). Within the MWCNT cluster, two sub-clusters were identified: 1) the cluster colored in pink, which included datasets from the bleomycin-induced injury and bacterial infection models; and 2) the blue cluster which consisted of the dataset from the ovalbumin sensitization model characteristic of a Th2 response. Both the pink and blue clusters contained experimental samples from studies of MWCNTs with different physic-chemical properties and included a range of doses. The clustering of the MWCNT-exposed samples within these sub-clusters was mainly associated with different post-exposure time points at which the analysis was conducted. The MWCNT data sets in the pink cluster alongside the bleomycin/bacterial studies were primarily from the 24 h post-exposure time point; whereas, those found in the blue cluster (together with Th2 response studies) were predominantly from the 14, 28 and 56 days post-exposure time points. A 3 day post-MWCNT exposure time point was also included in the meta-analysis. These datasets did not show distinct clusters and were scattered across the MWCNT cluster, with 4 of the 6 experimental groups clustering in pink with the bleomycin/bacteria models. The analysis suggests that the initial response to MWCNT administration is similar to lung responses observed following bacterial challenge or following exposure to a damaging substance like bleomycin; whereas, later responses to MWCNT are similar to Th2 response induced following exposure to allergens. Th2-induced gene expression of several cytokines, chemokines and growth factors is known to play a critical role in the progression of lung fibrosis [27], a lung disease observed at 14 or 28 days following exposures to MWCNTs [28]. Examining the genes that drive this clustering may provide insight into the biological processes engaged in these two distinct phases of the pulmonary responses to MWCNTs.

In general, the clusters showed clear associations between datasets from MWCNT-exposed lungs and lung disease models; however, some individual samples from the bleomycin injury, bacterial infection, nanoTiO_2_, and arc-stainless steel studies clustered under a separate branch at the distal end of the MWCNT cluster. The clustering of these samples may reflect differences in the individual animal responses. Similarly, several CB and nanoTiO_2_ groups including the only inhalation study describing responses to inhaled nanoTiO_2_ consisting of single dose and single post-exposure time point were clustered together in between the pink and the blue cluster. Although it is tempting to speculate that the route of exposure (inhalation vs instillation) may have something to do with this unique clustering in the case of the nanoTiO_2_ inhalation study group, detailed analysis of DEGs in the cluster did not reveal such unique features. More studies systematically investigating the responses following exposure to particles delivered via two different administration routes are required.

Additionally, some CB experimental groups were observed in the pink (bleomycin/bacteria) and the blue (Th2 response) sub-clusters. These findings imply that transcriptional responses to carbon-based nanoparticles (CB and MWCNTs) may share some similarities; however, we caution that the results of the CB-exposed samples are inconsistent. Transcriptional response to stainless steel also clustered separately. The closest nodes to stainless steel were acute exposure to bleomycin and acute exposures to nanoTiO_2_. Since only MWCNT datasets showed clear association with the lung disease models, the analysis described below focused on the pink and blue sub-clusters.

### GO functional analysis of DEGs intersecting bacteria/bleomycin (pink) and Th2 response (blue) clusters

**Fig. 2:**
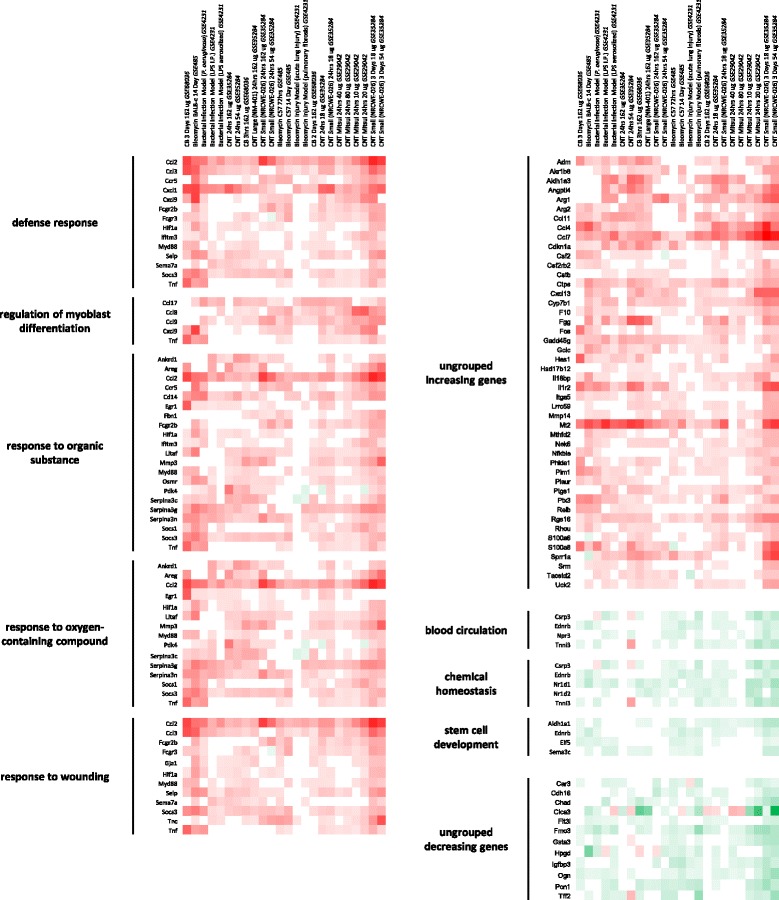
*The bacteria/bleomycin cluster was enriched in DEGs associated with defensive responses to various stimuli*. A short list of genes was generated for the bacteria/bleomycin cluster (highlighted in pink in Fig. 1). Genes that were differentially expressed in at least 50 % of the arrays that make up the cluster were included in the shortlist. The ClueGO app for Cytoscape software with GO Fusion was utilized to identify biological functions that were enriched in the shortlist and reduce any repetitive GOs. GOs were collapsed back to their parent GOs to further simplify data presentation. A heatmap was generated to visualize the cluster’s shortlist, organized by biological function

**Fig. 3:**
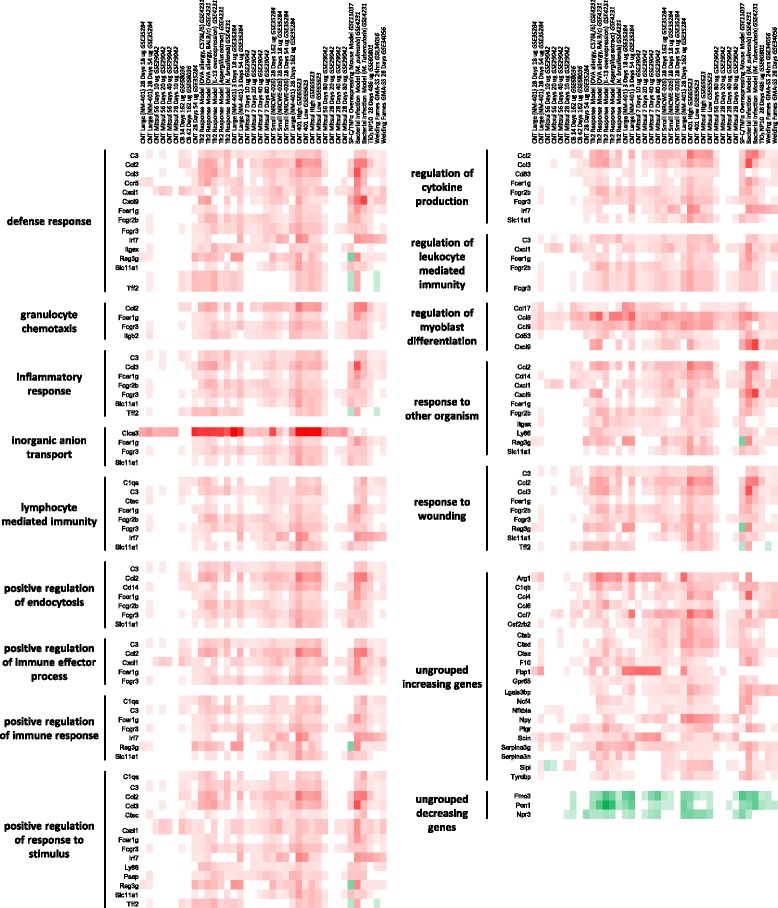
*Enrichment analysis of the biological functions in the Th2 cluster involved the immune response.* A short list of genes was generated for the Th2 response cluster (highlighted in blue in Fig. 1). Genes that were differentially expressed in at least 50 % of the arrays that make up the cluster were included in the shortlist. The ClueGO app for Cytoscape software with GO Fusion was utilized to identify biological functions that were enriched in the shortlist and reduce any repetitive GOs. GOs were further collapsed back to their parent GOs to further simplify data presentation. A heatmap was generated to visualize the cluster’s shortlist, organized by biological function

It is expected that the essential features of any biological response, including a disease phenotype, are overrepresented in the affected/exposed tissues relative to the unaffected/unexposed control tissues. To identify these transcriptional features we explored transcriptional similarities of the lung disease models and MWCNT-induced pulmonary responses, by investigating the genes in common with the various experimental conditions that made up the pink and blue sub-clusters. A shortlist of upregulated and downregulated genes was generated for each intersection, which consisted of genes that were significantly altered in at least 50 % of the datasets within that specific cluster. Gene expression fold changes were used to generate a heat map for each cluster (Figs. 2 and 3). The shortlist of genes in the bleomycin/bacterial cluster (pink) consisted of 79 upregulated and 21 downregulated genes (Fig. 2). The Th2 response cluster (blue) consisted of 53 upregulated and 3 downregulated genes (Fig. 3). There was a high degree of overlap in genes between the different biological functions identified.

In order to investigate the type of biological processes perturbed by the DEGs that are common in either the pink bleomycin/bacteria or the blue Th2 response sub-clusters, the short list of genes was analyzed using the Cytoscape app, ClueGo. This analysis takes into account the experimentally curated gene ontologies specific to biological processes. Because the pink cluster consists of acute post exposure time points and the blue cluster consists of longer post-exposure time points, this analysis effectively differentiates biological processes engaged in the early and late phases of the response to MWCNT, in addition to highlighting the processes that overlap with lung disease models.

### Biological processes/functions perturbed in bacteria/bleomycin cluster

The short list of DEGs from the pink cluster was enriched in host defense GOs and the response to specific stimuli (Fig. 2). Specifically, *defense response* (GO:0006952), which indicates that mechanisms involved in host-defense against invasive agents were engaged, and *response to wounding* (GO:0009611), implying that MWCNTs are damaging to the lung environment at early time points, were enriched. The other enriched biological processes included *response to organic substance* (GO:0010033) and *response to oxygen containing compound* (GO:1901700), suggesting a commonality between the response to this broad group of stimuli and MWCNTs. Five genes from the shortlist, *Ccl2*, *Hif1a*, *Myd88*, *Socs3*, and *Tnf*, were common to all of these GOs. In addition, *regulation of myoblast differentiation* (GO:0045661) was enriched, but its involvement in the pulmonary response to MWCNT is not clear. Forty-six of the upregulated genes were not associated with any enriched GOs in this analysis. Enrichment analysis of the downregulated genes identified *blood circulation* (GO:0008015), *chemical homeostasis* (GO:0048878), and *stem cell development* (GO:0048864) processes to be altered, which indicates that homeostatic processes like the circulation of blood and developmental processes may be disrupted in the lungs by acute MWCNT exposure as it is in bacterial infection and bleomycin exposure. Twelve of the downregulated genes could not be associated with any enriched GO.

### Biological processes/functions perturbed in Th2 response cluster

Similar to the pink cluster, the enriched GOs in the blue cluster also included in host-defense mechanisms (Fig. 3), such as *defense response* (GO:0006952), *inflammatory response* (GO:0006954), *granulocyte chemotaxis* (GO:0071621), *regulation of leukocyte mediated immunity* (GO:0002703), and *positive regulation of endocytosis* (GO:0045807). GOs related to a broad range of immune functions were also enriched, including *response to other organism* (GO:0051707), *positive regulation of immune response* (GO:0050778), and *regulation of cytokine production* (GO:0001817). In addition, GOs related to the adaptive immune response such as *lymphocyte mediated immunity* (GO:0002449) were also enriched. Adaptive immunity and *lymphocyte function* are key components that characterize the Th2 response and this GO represents an important overlap between MWCNT exposure and the Th2 response model. Upregulated genes associated with these immune response GOs included *C1qa*, *C3*, *Ccl2*, *Ccl3*, *Cxcl1*, *Cxcl9*, *Fcer1g*, *Fcgr2b*, *Fcgr3*, *Irf7*, and *Slc11a1*. The *inorganic anion transport* (GO:0015698) and *regulation of myoblast differentiation* (GO:0045661) ontologies were also enriched in this analysis, although their relevance to the response to MWCNT is less intuitive. The enrichment of *positive regulation of response to stimulus* (GO:0048584) and *response to wounding* (GO:0009611) was also observed, which suggested that damage to the pulmonary environment is activating repair mechanisms at early and late time points; however, a large number of adaptive immune mechanisms are engaged at later phases of the response to MWCNTs. Only 3 genes were down regulated in this shortlist and enrichment analysis of such a small list did not produce any meaningful results.

Visual inspection of the heat maps (Figs. 2 and 3) reveals a pattern that is consistent with a dose response in gene expression changes at later post-MWCNT exposure time points. Although the pink cluster showed similar transcription levels across all of the arrays (Fig. 2), gene expression patterns in the blue cluster revealed time and dose-dependence (Fig. 3). Specifically, samples exposed to lower doses (40 μg/mouse or less) MWCNTs analyzed at 56 days post-exposure showed less similarity in expression changes with the Th2 response model. In contrast, mice exposed to more than 40 μg/mouse showed a higher degree of similarity in transcriptional profiles to the Th2 response models at 3, 7 and 28 days post-exposure time points. Thus, there was a dose and time dependency in association of MWCNT samples with disease models.

### Analysis of upstream regulators controlling the perturbed processes in both bacteria/bleomycin and Th2 response clusters

**Fig. 4:**
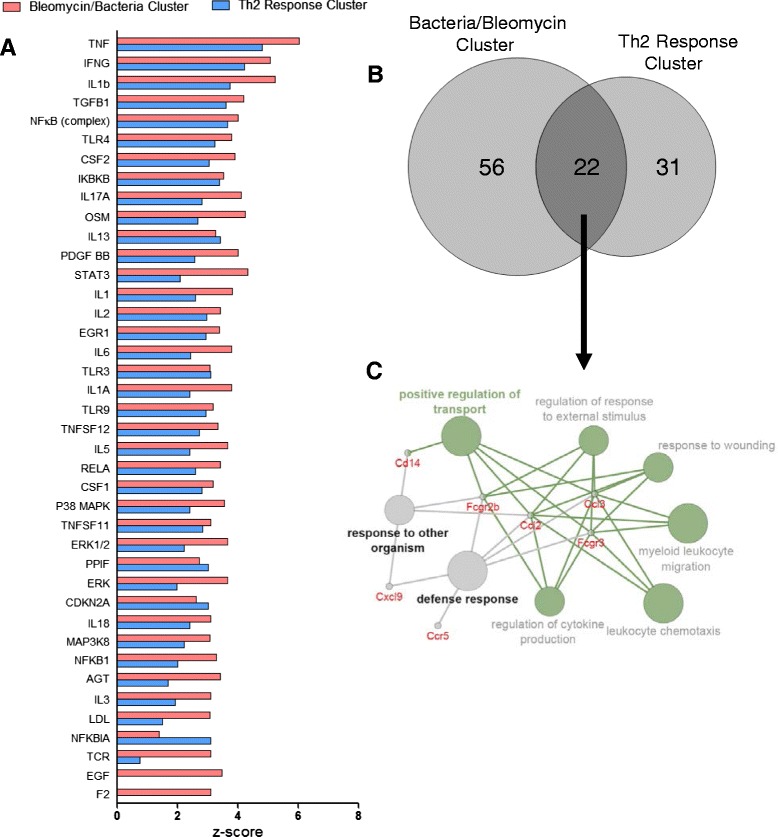
*Upstream regulator analysis identifies inflammatory mediators similar to both clusters.* **a** IPA software was used to identify the upstream regulators of the gene expression profile of the pink and blue clusters. **b** A Venn diagram was constructed to investigate the similarity between the genes that were increased in each of the shortlists, demonstrating the overlap between them. **c** The Cytoscape apps ClueGO and CluePedia created a visual network of the biological functions enriched in these overlapping genes and identified specific genes that are common to different biological functions. All but two of the GOs were grouped together based on similar gene expression patterns and are highlighted in green, with the *positive regulation of transport* GO being the most significantly enriched biological function in the group

IPA was used to identify the upstream transcriptional regulators potentially controlling the observed gene expression changes in the pink and blue clusters. Upstream regulators showing a Z-score above 2.0 are shown in Fig. 4a. Interestingly, a number of genes known to regulate inflammatory processes (*Tnf*, *Ifng*, and *Il1b*), and genes involved in regulating cell proliferation and growth (*Tgfb1* and *Csf2*)*,* had the highest Z-scores for both sub-clusters. This suggests that the two clusters are regulated by similar processes. A Venn analysis of the upregulated genes in the shortlists from both sub-clusters confirmed a total of 22 overlapping genes that were upregulated in both clusters (Fig. 4b). No such analysis could be conducted for the down regulated genes, as the blue cluster shortlist contained only 3 down regulated DEGs. Further analysis of the 22 overlapping genes using ClueGo/Clupedia network analysis showed that these genes are mainly associated with recruitment of leukocytes and were grouped under *positive regulation of transport* (GO:0051050), *leukocyte chemotaxis* (GO:0030595), *myeloid leukocyte migration* (GO:0097529), and *regulation of cytokine production* (GO:0001817) (Fig. 4c). The *Ccl2*, *Ccl3*, *Fcgr2b*, and *Fcgr3* genes were common to all GO categories. Collectively, this analysis suggests that inflammation and inflammation related genes govern the responses observed in the two sub-clusters (pink and blue). These results suggest that both phases of the response to MWCNT are driven by inflammatory mediators despite distinct clustering of the early and late phases of the MWCNT with different lung disease models, and that several of these mediators are continually expressed.

### Gene expression changes associated with lung fibrosis

The lung injury models including bleomycin, Th2 response-mediated allergy, and bacterial infection are all known to cause lung fibrosis [27]. Similarly, exposure to different variants of MWCNTs induces pulmonary fibrosis in several studies [28, 29]. In fact, lung fibrosis is a well-characterized adverse pulmonary outcome induced by MWCNTs. Thus, the short lists of intersecting genes were examined for their direct association with lung fibrosis.

**Fig. 5:**
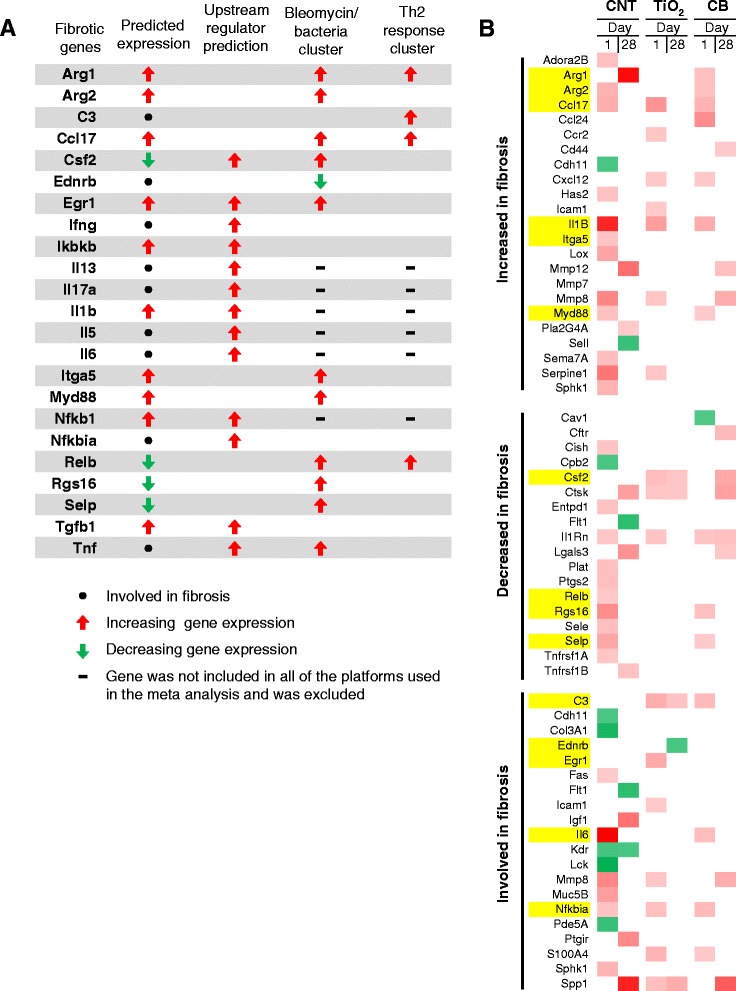
*The MWCNT sub-clusters include pro-fibrotic genes that can differentiate MWCNT from less fibrotic nanomaterials.* **a** A list of *fibrosis* genes was obtained from the IPA database and the overlapping genes shortlisted in the pink and blue clusters, as well as the genes identified as upstream regulators of the pink and blue cluster shortlists, are indicated. **b** The fibrosis gene list from IPA was also applied to gene shortlists from CNT, TiO_2_, and CB datasets that were included in this meta-analysis with the same dose and time points to show the differences in gene expression between MWCNTs and other less fibrotic nanomaterials. Genes from Fig. 5a are highlighted in yellow

Lung fibrosis has been extensively studied and several of the genes involved in this process have been identified. Using the IPA pathway and network database, a list of 105 genes associated with *fibrosis of the lung* network was created. Next, the gene lists from the pink and blue clusters, as well as genes identified in the upstream regulator analysis, were overlaid onto the IPA fibrosis network gene list. This analysis identified 23 specific genes associated with both clusters that are directly relevant to the progression of lung fibrosis (Fig. 5a). Of particular interest were *Arg1*, *C3*, and *Ccl17*, genes which were increased at 7, 28, and 56 days post-exposure time points in MWCNT studies, when fibrosis is observed histologically in this model [8]. Interestingly, *Arg1*, *C3*, and *Ccl17* are all genes considered to be biomarkers of alternatively activated macrophages, a cell type that requires Th2-regulated cytokines for its differentiation, and that also have been implicated in the progression of pulmonary fibrosis [30, 31].

Meta-analysis of the gene expression data derived from various studies identified MWCNTs as potential fibrosis-inducing substances that could possibly mediate their fibrotic response *via* the Th2-mediated signaling. However, given the variability in the study designs, doses used and statistical normalization processes employed, the genes in the cluster shortlists would not be expected to include all of the potential pro-fibrotic genes induced by MWCNT exposure. Although, the cluster analysis may be used to identify a portion of the biological response that is involved in fibrotic pathogenesis, it is likely that additional genes are involved. As such, several pro-fibrotic genes that may have been altered in response to nanoTiO_2_ and CB exposures may not have been measurably altered in this study, this likely resulted in differential grouping of these substances.

To investigate this hypothesis, the list of IPA’s *fibrosis of the lung* disease network genes was overlaid onto the day 1 and day 28 DEG lists of mouse lungs exposed to 162 μg of MWCNTs, nanoTiO_2,_ or CB (from studies that were included in the meta-analysis) [8, 10, 11]. These genes were divided into three groups depending on whether the genes are expected to increase fibrosis, decrease fibrosis, or if the direction of change is uncertain (Fig. 5b). The genes highlighted in yellow were the ones identified through the cluster analysis (Fig. 5a). This analysis revealed additional genes from the MWCNT datasets associated with fibrosis but not many genes from the nanoTiO_2_ and CB studies. This expanded list of genes included *Mmp12*, *Spp1*, *Pla2g4a*, *Igf1*, and *Ptgir* which were expressed at greater levels in MWCNT exposed lungs at 28 days post-exposure.

## Discussion

Microarray technology has been extensively used to investigate the relationship between gene expression profiles and molecular mechanisms underlying diseases. In this study, we compared pulmonary gene expression profiles derived from mice exposed to different ENMs to those induced by chemical substances or pathogens causing lung diseases. Our meta-analysis demonstrates a feasible approach for the application of global gene expression profiling to identify the potential of ENMs to cause pulmonary pathogenesis. By using a variety of lung models of inflammatory diseases, we identified common transcriptional changes between lung diseases and ENM-induced lung responses. This allowed us to bin MWCNTs as potentially disease-causing ENMs separately from the nanoTiO_2_ and CB, as ENMs not associated with lung disease at the concentrations and time points examined in this study. This work also revealed potential mechanisms by which MWCNTs induce lung fibrosis, and identified a suite of marker genes that may serve as predictors of lung fibrosis.

Several studies argue that inflammation does not play a role in pulmonary fibrosis in general [32, 33]. This is mainly due to the lack of an influx of inflammatory cells observed in patients presenting with lung pathology in parallel with the lack of efficacy of immunosuppressive drugs in fibrosis patients [34, 35]. In line with this argument, in MWCNT-exposed mice presenting with the fibrotic phenotype (studies included in the analyses), BAL cellular influx recedes (but does not completely reverse) with time and fibrotic lesions do not present inflammatory characteristics [8]. However, the gene expression data shown here suggest otherwise and provide clues as to how the process of inflammation may participate in the fibrotic pathology. The meta-analysis showed that pulmonary gene expression signatures induced by MWCNTs were similar to patterns of bacterial, bleomycin, and Th2-mediated allergic response models that induce robust pulmonary inflammation, and revealed altered gene expression of a large suite of inflammatory cytokines and chemokines across the acute and sub chronic post-exposure time points. The results also showed that the upstream regulators governing the altered gene expression profiles involved the transcriptional factors responsible for regulating inflammation.

In addition to cytokines, genes involved in alternatively activated (M2) macrophages resulting from polarized T-cell response (eg. *Arg1*, *C3*, and *Ccl17*) were upregulated in MWCNT-exposed lung datasets. M1 and M2 macrophage types are involved in mediating pulmonary inflammatory responses at different stages of the response [36]. M1 macrophages kill invading pathogens or injured cells and produce pro-inflammatory cytokines [36]. In contrast, activated M2 macrophages secrete anti-inflammatory cytokines, regulate M1-mediated inflammatory responses, aid in clearance of cellular debris, and promote angiogenesis, tissue remodeling, and repair [36]. Upregulation of *Arg1*, *C3*, and *Ccl17* potentially results in the activation of the M2 phenotype, and simply reflects the transition to a different functional state (from innate immune to adaptive response) in cells battling persistent injury inflicted by residual MWCNTs. This was also evident by the temporal nature of the association between the MWCNT-induced lung responses and disease models; early responses (24 h, 3days) had transcriptional profiles that were consistent with bacterial- or bleomycin-induced injury responses, and the later responses (28days) suggested an association with Th2-mediated response, reflecting a transition from early innate immune response to later adaptive immune responses, respectively. Thus, profuse inflammation, continuing injury, and tipping of the delicate balance between pro- and anti-inflammatory mediators results in the activation of alternate cell types and secretion of pro-fibrotic molecules which creates a microenvironment conducive for the development of lung fibrosis. The inflammatory process has been implicated in lung fibrosis induced by crystalline silica and asbestos [37], both of which are shown to accumulate in alveolar macrophages resulting in the activation of an inflammatory cascade that eventually results in lung fibrosis. MWCNTs are suggested to behave like asbestos due to their fiber like structures [38]. Collectively, these results suggest an important role for inflammation in ensuing lung fibrosis induced by MWCNTs.

Although nanoTiO_2_ and CB exposure also induce lung inflammation and alter the expression of several pro-inflammatory genes [39], mice exposed to these ENMs did not show a clear association with the disease models. There are many differences in the characteristics of inflammation produced by nanoTiO_2_ and CB, including: 1) CB/nanoTiO_2_ exposure causes significantly less inflammation than MWCNT in mice exposed at similar exposure doses and sampling regimens; 2) nanoTiO_2_and CB induced inflammation is predominantly neutrophilic; whereas, MWCNT induced inflammation involves multiple cell types; 3) with the exception of the highest doses examined, BAL cellular influx significantly declines 28days post-exposure following nanoTiO_2_ and CB exposure, it is less reduced in mice exposed to MWCNT; 4) gene expression patterns in nanoTiO_2_ or CB exposed samples are mainly associated with acute innate response; and, 5) the magnitude of expression change for any given gene is relatively lower for nanoTiO_2_ or CB exposed samples compared with MWCNTs and the majority of these changes are reversed by 28 days post-exposure [8, 10, 11]. Although some CB samples were interspersed with the disease models, the response varied within specific experimental conditions and lacked consistency. Neither nanoTiO_2_ nor CB have been shown to induce lung fibrosis at the doses or time points analyzed in this study.

The other interesting observation, though subtle, was that the experimental conditions from the welding fumes study clustered differently in a manner that seemed to depend on their lung administration methods (aspiration versus inhalation). However, the two studies were conducted at different times and varied in the experimental design including the doses administered. In the context of ENMs, it can be expected that lung responses to ENMs introduced via inhalation, aspiration or intratracheal deposition may be different because of the differences in the deposited dose and ENM distribution in lungs. However, we have previously shown that lung inflammatory responses to surface-coated TiO_2_NPs deposited in mouse lungs via intratracheal instillation or inhalation are similar [11]. Jackson et al did not observe differences in the lung distribution or effects in mice exposed to carbon black particles via intratracheal instillation or inhalation [40]. Future studies directly comparing the pulmonary toxicogenomics responses from mice exposed to ENMs via inhalation or other lung administration routes are warranted. Currently, such a comparison is not possible as lung toxicogenomics data from inhalation models are not available.

Other groups have employed global gene expression profiling to identify signatures predictive of lung cancer for ENMs. Pacurari et al*.* showed changes in the expression of a select set of lung cancer biomarker genes following exposure to MWCNTs and suggested a link between MWCNT-induced lung inflammation, fibrosis, and cancer [41]. A follow up study by Guo et al*.* conducted genome-wide gene expression profiling of MWCNT-exposed mouse lungs and compared the gene expression profiles to human lungs derived from lung cancer patients to predict lung cancer progression by correlating mouse gene expression data with expression in patient lungs [14]. A recent study by Sargent et al showed that Mitsui-7, a rigid, rod-like fiber acts as a cancer promoter; however, there is no current evidence to support the occurrence of lung cancer following exposure to all types of MWCNTs [42].

In addition to lung cancer and fibrosis, MWCNTs may also contribute to other lung diseases, though little data exist to support this possibility. Consistent with the findings of our meta-analysis demonstrating similarities in the transcriptional response of MWCNT-exposed mice and Th2 disease models, MWCNT have been shown to stimulate and exacerbate allergic airways [28, 43–45]. These animal models demonstrate an increase in allergic inflammation, mucus hyperplasia, and fibrotic airway when MWCNT are administered along with an allergen or surrogate allergen. These observations from allergy models highlight an important consideration for the toxic properties of MWCNTs as these materials may play a synergistic role with other lung pathologies. With respect to pulmonary infections, MWCNT administration is associated with increased infectivity of the influenza virus, implying a suppression of certain host defense mechanisms after MWCNT exposure [46]. Increased mucus production can exacerbate diseases with pathologies that include airway obstruction by further decreasing airflow. MWCNTs have been shown to increase mucus production [47], which has the potential to exacerbate asthma and Chronic Obstructive Pulmonary Disease. The current analysis characterizes the biological response to MWCNTs, but the extent of the toxic outcomes of this response may not be fully realized in isolation. The effects induced in a MWCNT exposed lung in the context of other diseases should be considered to fully appreciate the breadth of MWCNT-induced pathology.

## Conclusions

**Fig. 6:**
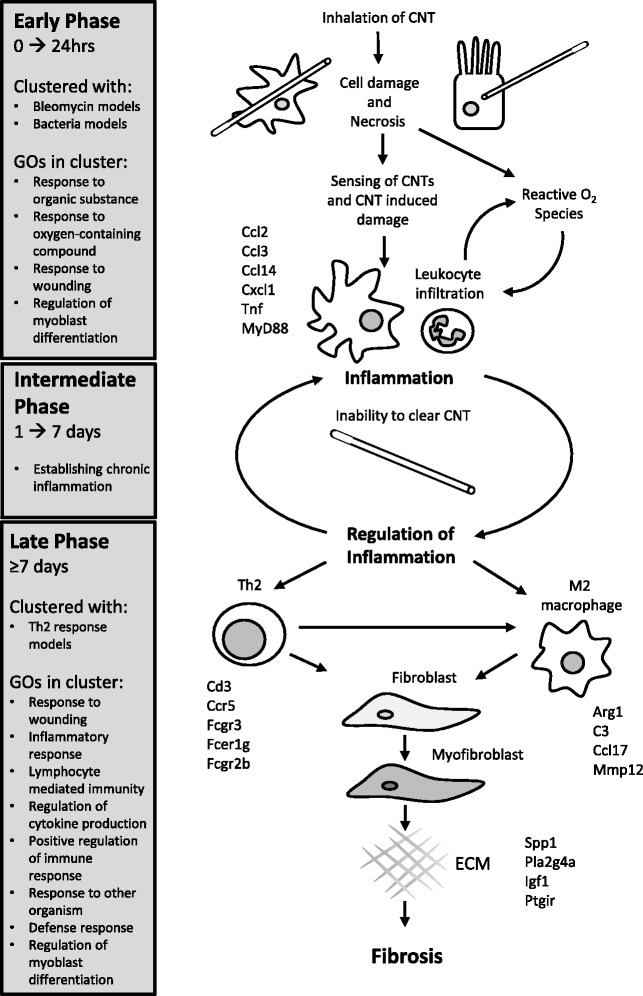
*The cluster analysis indicates a potential pathway for MWCNT-induced fibrosis based on two distinct phases.* In summary of the results of the cluster analysis, a pathway can be constructed representing the two phases of the lung response to the MWCNT with relation to the disease models that share similar gene expression. The genes and GOs identified in the meta-analysis are organized into the events along the pathway where they are likely involved. Due to the lack of clustering, the response to nanoTiO_2_ and CB could not be similarly summarized

In summary, the findings of our meta-analysis identified genes and biological processes involved in two distinct phases of the pulmonary response to MWCNTs. This response was characterized by its similarity to existing models of lung disease and was distinct from the response elicited by nanoTiO_2_ and CB nanoparticles. Some of the key genes and GOs identified in this study are depicted in Fig. 6, which describes a possible mechanism of pathology. Inflammation and Th2 responses have previously been implicated in the progression of pulmonary fibrosis [27], and the current analysis also highlights the potential importance of alternatively activated macrophages in this process. The cluster analysis provided new insights into the mechanisms of disease engaged by a nanomaterial capable of inducing fibrosis and highlighted how these mechanisms are not engaged by nanoparticles that do not induce fibrosis.

The systematic and comparative meta-profiling of a small set of publicly available microarray datasets enabled characterization of a transcriptional signature that is shared between various lung disease models and one that is essential to MWCNT-induced lung fibrosis. The observation of generalized activation of specific gene mechanisms regardless of their specific properties suggests that these genes and mechanisms are strongly associated with lung fibrosis. As the public repository of transcriptome data becomes populated, the simple framework described here for comparing and assessing the intersections of multiple gene expression patterns from different datasets will enable effective differentiation between the disease inducing and benign ENMs, and aid in prioritizing of ENMs for further investigation. It is important to note that the approach proposed is not an *in silico* computational approach and requires tissue-specific gene expression profiling following exposure to novel ENMs. However, it is proposed that the strategy (short term *in vivo* tissue transcriptomics combined with meta-analysis) can be used as an effective first tier screening of new ENMs that exhibit higher potential to induce pulmonary pathology. The study results presented here reflect lung responses to ENMs in mouse model. However, many of the significantly perturbed pro-fibrotic genes and pathways identified are known to be associated with lung fibrosis in humans. Conditional to appropriate validation, some of the genes may potentially be used to support future biomonitoring and surveillance efforts for ENMs.

## Ethic approval and consent to participate

All studies selected for the analysis obtained proper ethics approval from their respective institutions animal ethics boards. No animal experiments were conducted in addition to the previously published studies.

## Availability of data and material

All experimental data is available through GEO under the GSE identification numbers outlined in Table 1.

## Additional file

Additional file 1: Table S1. A list of 2334 differentially expressed genes that were consistent to all of the microarray platforms employed in the studies included in the meta-analysis. (PPTX 123 kb)

## Abbreviations

BAL: bronchoalveolar lavage
CB: carbon black
CNTs: carbon nanotubes
DEGs: differentially expressed genes
ENMs: engineered nanomaterials
GEO: Gene Expression Omnibus
GOs: gene ontologies
IPA: Ingenuity Pathway Analysis
LPS: lipopolysaccharide
MWCNTs: multi-walled carbon nanotubes
NM: nanomaterial
TiO_2_: titanium dioxide
