# Supplementary material for: Meta-analysis of transcriptomic responses as a means to identify pulmonary disease outcomes for engineered nanomaterials
Source: Part Fibre Toxicol. 2016 May 11;13:25. doi: 10.1186/s12989-016-0137-5 (PMC4865099; doi:10.1186/s12989-016-0137-5)
Supplement: Additional file 1: Table S1. — A list of 2334 differentially expressed genes that were consistent to all of the microarray platforms employed in the studies included in the meta-analysis. (PPTX 123 kb) [file 12989_2016_137_MOESM1_ESM.pptx]

## Slide 1
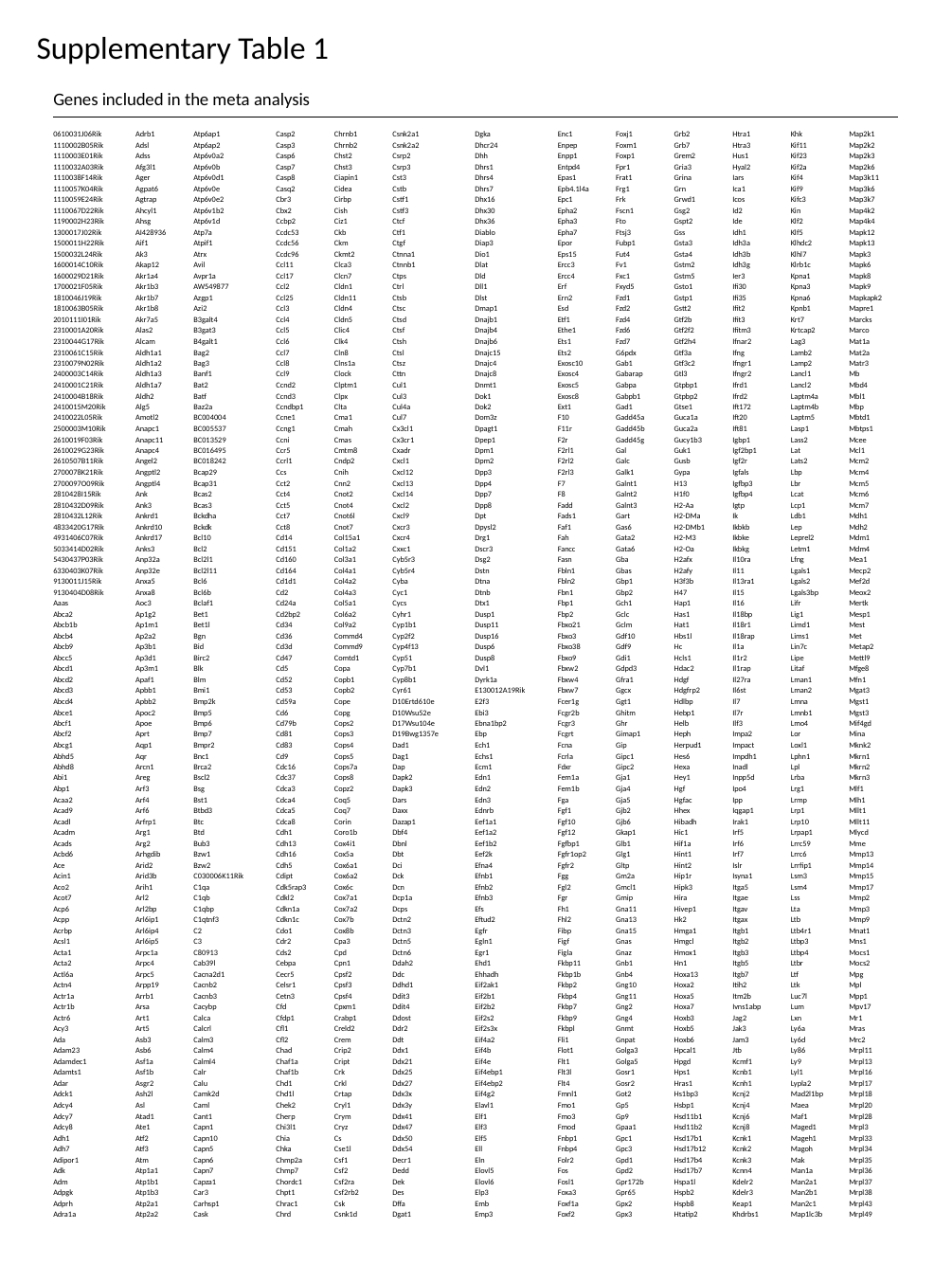

Supplementary Table 1
| Genes included in the meta analysis | | | | | | | | | | | | | | | | | | | | | | | | |
| --- | --- | --- | --- | --- | --- | --- | --- | --- | --- | --- | --- | --- | --- | --- | --- | --- | --- | --- | --- | --- | --- | --- | --- | --- |
| | | | | | | | | | | | | | | | | | | | | | | | | |
| 0610031J06Rik | | Adrb1 | | Atp6ap1 | | Casp2 | | Chrnb1 | | Csnk2a1 | | Dgka | | Enc1 | | Foxj1 | | Grb2 | | Htra1 | | Khk | | Map2k1 |
| 1110002B05Rik | | Adsl | | Atp6ap2 | | Casp3 | | Chrnb2 | | Csnk2a2 | | Dhcr24 | | Enpep | | Foxm1 | | Grb7 | | Htra3 | | Kif11 | | Map2k2 |
| 1110003E01Rik | | Adss | | Atp6v0a2 | | Casp6 | | Chst2 | | Csrp2 | | Dhh | | Enpp1 | | Foxp1 | | Grem2 | | Hus1 | | Kif23 | | Map2k3 |
| 1110032A03Rik | | Afg3l1 | | Atp6v0b | | Casp7 | | Chst3 | | Csrp3 | | Dhrs1 | | Entpd4 | | Fpr1 | | Gria3 | | Hyal2 | | Kif2a | | Map2k6 |
| 1110038F14Rik | | Ager | | Atp6v0d1 | | Casp8 | | Ciapin1 | | Cst3 | | Dhrs4 | | Epas1 | | Frat1 | | Grina | | Iars | | Kif4 | | Map3k11 |
| 1110057K04Rik | | Agpat6 | | Atp6v0e | | Casq2 | | Cidea | | Cstb | | Dhrs7 | | Epb4.1l4a | | Frg1 | | Grn | | Ica1 | | Kif9 | | Map3k6 |
| 1110059E24Rik | | Agtrap | | Atp6v0e2 | | Cbr3 | | Cirbp | | Cstf1 | | Dhx16 | | Epc1 | | Frk | | Grwd1 | | Icos | | Kifc3 | | Map3k7 |
| 1110067D22Rik | | Ahcyl1 | | Atp6v1b2 | | Cbx2 | | Cish | | Cstf3 | | Dhx30 | | Epha2 | | Fscn1 | | Gsg2 | | Id2 | | Kin | | Map4k2 |
| 1190002H23Rik | | Ahsg | | Atp6v1d | | Ccbp2 | | Ciz1 | | Ctcf | | Dhx36 | | Epha3 | | Fto | | Gspt2 | | Ide | | Klf2 | | Map4k4 |
| 1300017J02Rik | | AI428936 | | Atp7a | | Ccdc53 | | Ckb | | Ctf1 | | Diablo | | Epha7 | | Ftsj3 | | Gss | | Idh1 | | Klf5 | | Mapk12 |
| 1500011H22Rik | | Aif1 | | Atpif1 | | Ccdc56 | | Ckm | | Ctgf | | Diap3 | | Epor | | Fubp1 | | Gsta3 | | Idh3a | | Klhdc2 | | Mapk13 |
| 1500032L24Rik | | Ak3 | | Atrx | | Ccdc96 | | Ckmt2 | | Ctnna1 | | Dio1 | | Eps15 | | Fut4 | | Gsta4 | | Idh3b | | Klhl7 | | Mapk3 |
| 1600014C10Rik | | Akap12 | | Avil | | Ccl11 | | Clca3 | | Ctnnb1 | | Dlat | | Ercc3 | | Fv1 | | Gstm2 | | Idh3g | | Klrb1c | | Mapk6 |
| 1600029D21Rik | | Akr1a4 | | Avpr1a | | Ccl17 | | Clcn7 | | Ctps | | Dld | | Ercc4 | | Fxc1 | | Gstm5 | | Ier3 | | Kpna1 | | Mapk8 |
| 1700021F05Rik | | Akr1b3 | | AW549877 | | Ccl2 | | Cldn1 | | Ctrl | | Dll1 | | Erf | | Fxyd5 | | Gsto1 | | Ifi30 | | Kpna3 | | Mapk9 |
| 1810046J19Rik | | Akr1b7 | | Azgp1 | | Ccl25 | | Cldn11 | | Ctsb | | Dlst | | Ern2 | | Fzd1 | | Gstp1 | | Ifi35 | | Kpna6 | | Mapkapk2 |
| 1810063B05Rik | | Akr1b8 | | Azi2 | | Ccl3 | | Cldn4 | | Ctsc | | Dmap1 | | Esd | | Fzd2 | | Gstt2 | | Ifit2 | | Kpnb1 | | Mapre1 |
| 2010111I01Rik | | Akr7a5 | | B3galt4 | | Ccl4 | | Cldn5 | | Ctsd | | Dnajb1 | | Etf1 | | Fzd4 | | Gtf2b | | Ifit3 | | Krt7 | | Marcks |
| 2310001A20Rik | | Alas2 | | B3gat3 | | Ccl5 | | Clic4 | | Ctsf | | Dnajb4 | | Ethe1 | | Fzd6 | | Gtf2f2 | | Ifitm3 | | Krtcap2 | | Marco |
| 2310044G17Rik | | Alcam | | B4galt1 | | Ccl6 | | Clk4 | | Ctsh | | Dnajb6 | | Ets1 | | Fzd7 | | Gtf2h4 | | Ifnar2 | | Lag3 | | Mat1a |
| 2310061C15Rik | | Aldh1a1 | | Bag2 | | Ccl7 | | Cln8 | | Ctsl | | Dnajc15 | | Ets2 | | G6pdx | | Gtf3a | | Ifng | | Lamb2 | | Mat2a |
| 2310079N02Rik | | Aldh1a2 | | Bag3 | | Ccl8 | | Clns1a | | Ctsz | | Dnajc4 | | Exosc10 | | Gab1 | | Gtf3c2 | | Ifngr1 | | Lamp2 | | Matr3 |
| 2400003C14Rik | | Aldh1a3 | | Banf1 | | Ccl9 | | Clock | | Cttn | | Dnajc8 | | Exosc4 | | Gabarap | | Gtl3 | | Ifngr2 | | Lancl1 | | Mb |
| 2410001C21Rik | | Aldh1a7 | | Bat2 | | Ccnd2 | | Clptm1 | | Cul1 | | Dnmt1 | | Exosc5 | | Gabpa | | Gtpbp1 | | Ifrd1 | | Lancl2 | | Mbd4 |
| 2410004B18Rik | | Aldh2 | | Batf | | Ccnd3 | | Clpx | | Cul3 | | Dok1 | | Exosc8 | | Gabpb1 | | Gtpbp2 | | Ifrd2 | | Laptm4a | | Mbl1 |
| 2410015M20Rik | | Alg5 | | Baz2a | | Ccndbp1 | | Clta | | Cul4a | | Dok2 | | Ext1 | | Gad1 | | Gtse1 | | Ift172 | | Laptm4b | | Mbp |
| 2410022L05Rik | | Amotl2 | | BC004004 | | Ccne1 | | Cma1 | | Cul7 | | Dom3z | | F10 | | Gadd45a | | Guca1a | | Ift20 | | Laptm5 | | Mbtd1 |
| 2500003M10Rik | | Anapc1 | | BC005537 | | Ccng1 | | Cmah | | Cx3cl1 | | Dpagt1 | | F11r | | Gadd45b | | Guca2a | | Ift81 | | Lasp1 | | Mbtps1 |
| 2610019F03Rik | | Anapc11 | | BC013529 | | Ccni | | Cmas | | Cx3cr1 | | Dpep1 | | F2r | | Gadd45g | | Gucy1b3 | | Igbp1 | | Lass2 | | Mcee |
| 2610029G23Rik | | Anapc4 | | BC016495 | | Ccr5 | | Cmtm8 | | Cxadr | | Dpm1 | | F2rl1 | | Gal | | Guk1 | | Igf2bp1 | | Lat | | Mcl1 |
| 2610507B11Rik | | Angel2 | | BC018242 | | Ccrl1 | | Cndp2 | | Cxcl1 | | Dpm2 | | F2rl2 | | Galc | | Gusb | | Igf2r | | Lats2 | | Mcm2 |
| 2700078K21Rik | | Angptl2 | | Bcap29 | | Ccs | | Cnih | | Cxcl12 | | Dpp3 | | F2rl3 | | Galk1 | | Gypa | | Igfals | | Lbp | | Mcm4 |
| 2700097O09Rik | | Angptl4 | | Bcap31 | | Cct2 | | Cnn2 | | Cxcl13 | | Dpp4 | | F7 | | Galnt1 | | H13 | | Igfbp3 | | Lbr | | Mcm5 |
| 2810428I15Rik | | Ank | | Bcas2 | | Cct4 | | Cnot2 | | Cxcl14 | | Dpp7 | | F8 | | Galnt2 | | H1f0 | | Igfbp4 | | Lcat | | Mcm6 |
| 2810432D09Rik | | Ank3 | | Bcas3 | | Cct5 | | Cnot4 | | Cxcl2 | | Dpp8 | | Fadd | | Galnt3 | | H2-Aa | | Igtp | | Lcp1 | | Mcm7 |
| 2810432L12Rik | | Ankrd1 | | Bckdha | | Cct7 | | Cnot6l | | Cxcl9 | | Dpt | | Fads1 | | Gart | | H2-DMa | | Ik | | Ldb1 | | Mdh1 |
| 4833420G17Rik | | Ankrd10 | | Bckdk | | Cct8 | | Cnot7 | | Cxcr3 | | Dpysl2 | | Faf1 | | Gas6 | | H2-DMb1 | | Ikbkb | | Lep | | Mdh2 |
| 4931406C07Rik | | Ankrd17 | | Bcl10 | | Cd14 | | Col15a1 | | Cxcr4 | | Drg1 | | Fah | | Gata2 | | H2-M3 | | Ikbke | | Leprel2 | | Mdm1 |
| 5033414D02Rik | | Anks3 | | Bcl2 | | Cd151 | | Col1a2 | | Cxxc1 | | Dscr3 | | Fancc | | Gata6 | | H2-Oa | | Ikbkg | | Letm1 | | Mdm4 |
| 5430437P03Rik | | Anp32a | | Bcl2l1 | | Cd160 | | Col3a1 | | Cyb5r3 | | Dsg2 | | Fasn | | Gba | | H2afx | | Il10ra | | Lfng | | Mea1 |
| 6330403K07Rik | | Anp32e | | Bcl2l11 | | Cd164 | | Col4a1 | | Cyb5r4 | | Dstn | | Fbln1 | | Gbas | | H2afy | | Il11 | | Lgals1 | | Mecp2 |
| 9130011J15Rik | | Anxa5 | | Bcl6 | | Cd1d1 | | Col4a2 | | Cyba | | Dtna | | Fbln2 | | Gbp1 | | H3f3b | | Il13ra1 | | Lgals2 | | Mef2d |
| 9130404D08Rik | | Anxa8 | | Bcl6b | | Cd2 | | Col4a3 | | Cyc1 | | Dtnb | | Fbn1 | | Gbp2 | | H47 | | Il15 | | Lgals3bp | | Meox2 |
| Aaas | | Aoc3 | | Bclaf1 | | Cd24a | | Col5a1 | | Cycs | | Dtx1 | | Fbp1 | | Gch1 | | Hap1 | | Il16 | | Lifr | | Mertk |
| Abca2 | | Ap1g2 | | Bet1 | | Cd2bp2 | | Col6a2 | | Cyhr1 | | Dusp1 | | Fbp2 | | Gclc | | Has1 | | Il18bp | | Lig1 | | Mesp1 |
| Abcb1b | | Ap1m1 | | Bet1l | | Cd34 | | Col9a2 | | Cyp1b1 | | Dusp11 | | Fbxo21 | | Gclm | | Hat1 | | Il18r1 | | Limd1 | | Mest |
| Abcb4 | | Ap2a2 | | Bgn | | Cd36 | | Commd4 | | Cyp2f2 | | Dusp16 | | Fbxo3 | | Gdf10 | | Hbs1l | | Il18rap | | Lims1 | | Met |
| Abcb9 | | Ap3b1 | | Bid | | Cd3d | | Commd9 | | Cyp4f13 | | Dusp6 | | Fbxo38 | | Gdf9 | | Hc | | Il1a | | Lin7c | | Metap2 |
| Abcc5 | | Ap3d1 | | Birc2 | | Cd47 | | Comtd1 | | Cyp51 | | Dusp8 | | Fbxo9 | | Gdi1 | | Hcls1 | | Il1r2 | | Lipe | | Mettl9 |
| Abcd1 | | Ap3m1 | | Blk | | Cd5 | | Copa | | Cyp7b1 | | Dvl1 | | Fbxw2 | | Gdpd3 | | Hdac2 | | Il1rap | | Litaf | | Mfge8 |
| Abcd2 | | Apaf1 | | Blm | | Cd52 | | Copb1 | | Cyp8b1 | | Dyrk1a | | Fbxw4 | | Gfra1 | | Hdgf | | Il27ra | | Lman1 | | Mfn1 |
| Abcd3 | | Apbb1 | | Bmi1 | | Cd53 | | Copb2 | | Cyr61 | | E130012A19Rik | | Fbxw7 | | Ggcx | | Hdgfrp2 | | Il6st | | Lman2 | | Mgat3 |
| Abcd4 | | Apbb2 | | Bmp2k | | Cd59a | | Cope | | D10Ertd610e | | E2f3 | | Fcer1g | | Ggt1 | | Hdlbp | | Il7 | | Lmna | | Mgst1 |
| Abce1 | | Apoc2 | | Bmp5 | | Cd6 | | Copg | | D10Wsu52e | | Ebi3 | | Fcgr2b | | Ghitm | | Hebp1 | | Il7r | | Lmnb1 | | Mgst3 |
| Abcf1 | | Apoe | | Bmp6 | | Cd79b | | Cops2 | | D17Wsu104e | | Ebna1bp2 | | Fcgr3 | | Ghr | | Helb | | Ilf3 | | Lmo4 | | Mif4gd |
| Abcf2 | | Aprt | | Bmp7 | | Cd81 | | Cops3 | | D19Bwg1357e | | Ebp | | Fcgrt | | Gimap1 | | Heph | | Impa2 | | Lor | | Mina |
| Abcg1 | | Aqp1 | | Bmpr2 | | Cd83 | | Cops4 | | Dad1 | | Ech1 | | Fcna | | Gip | | Herpud1 | | Impact | | Loxl1 | | Mknk2 |
| Abhd5 | | Aqr | | Bnc1 | | Cd9 | | Cops5 | | Dag1 | | Echs1 | | Fcrla | | Gipc1 | | Hes6 | | Impdh1 | | Lphn1 | | Mkrn1 |
| Abhd8 | | Arcn1 | | Brca2 | | Cdc16 | | Cops7a | | Dap | | Ecm1 | | Fdxr | | Gipc2 | | Hexa | | Inadl | | Lpl | | Mkrn2 |
| Abi1 | | Areg | | Bscl2 | | Cdc37 | | Cops8 | | Dapk2 | | Edn1 | | Fem1a | | Gja1 | | Hey1 | | Inpp5d | | Lrba | | Mkrn3 |
| Abp1 | | Arf3 | | Bsg | | Cdca3 | | Copz2 | | Dapk3 | | Edn2 | | Fem1b | | Gja4 | | Hgf | | Ipo4 | | Lrg1 | | Mlf1 |
| Acaa2 | | Arf4 | | Bst1 | | Cdca4 | | Coq5 | | Dars | | Edn3 | | Fga | | Gja5 | | Hgfac | | Ipp | | Lrmp | | Mlh1 |
| Acad9 | | Arf6 | | Btbd3 | | Cdca5 | | Coq7 | | Daxx | | Ednrb | | Fgf1 | | Gjb2 | | Hhex | | Iqgap1 | | Lrp1 | | Mllt1 |
| Acadl | | Arfrp1 | | Btc | | Cdca8 | | Corin | | Dazap1 | | Eef1a1 | | Fgf10 | | Gjb6 | | Hibadh | | Irak1 | | Lrp10 | | Mllt11 |
| Acadm | | Arg1 | | Btd | | Cdh1 | | Coro1b | | Dbf4 | | Eef1a2 | | Fgf12 | | Gkap1 | | Hic1 | | Irf5 | | Lrpap1 | | Mlycd |
| Acads | | Arg2 | | Bub3 | | Cdh13 | | Cox4i1 | | Dbnl | | Eef1b2 | | Fgfbp1 | | Glb1 | | Hif1a | | Irf6 | | Lrrc59 | | Mme |
| Acbd6 | | Arhgdib | | Bzw1 | | Cdh16 | | Cox5a | | Dbt | | Eef2k | | Fgfr1op2 | | Glg1 | | Hint1 | | Irf7 | | Lrrc6 | | Mmp13 |
| Ace | | Arid2 | | Bzw2 | | Cdh5 | | Cox6a1 | | Dci | | Efna4 | | Fgfr2 | | Gltp | | Hint2 | | Islr | | Lrrfip1 | | Mmp14 |
| Acin1 | | Arid3b | | C030006K11Rik | | Cdipt | | Cox6a2 | | Dck | | Efnb1 | | Fgg | | Gm2a | | Hip1r | | Isyna1 | | Lsm3 | | Mmp15 |
| Aco2 | | Arih1 | | C1qa | | Cdk5rap3 | | Cox6c | | Dcn | | Efnb2 | | Fgl2 | | Gmcl1 | | Hipk3 | | Itga5 | | Lsm4 | | Mmp17 |
| Acot7 | | Arl2 | | C1qb | | Cdkl2 | | Cox7a1 | | Dcp1a | | Efnb3 | | Fgr | | Gmip | | Hira | | Itgae | | Lss | | Mmp2 |
| Acp6 | | Arl2bp | | C1qbp | | Cdkn1a | | Cox7a2 | | Dcps | | Efs | | Fh1 | | Gna11 | | Hivep1 | | Itgav | | Lta | | Mmp3 |
| Acpp | | Arl6ip1 | | C1qtnf3 | | Cdkn1c | | Cox7b | | Dctn2 | | Eftud2 | | Fhl2 | | Gna13 | | Hk2 | | Itgax | | Ltb | | Mmp9 |
| Acrbp | | Arl6ip4 | | C2 | | Cdo1 | | Cox8b | | Dctn3 | | Egfr | | Fibp | | Gna15 | | Hmga1 | | Itgb1 | | Ltb4r1 | | Mnat1 |
| Acsl1 | | Arl6ip5 | | C3 | | Cdr2 | | Cpa3 | | Dctn5 | | Egln1 | | Figf | | Gnas | | Hmgcl | | Itgb2 | | Ltbp3 | | Mns1 |
| Acta1 | | Arpc1a | | C80913 | | Cds2 | | Cpd | | Dctn6 | | Egr1 | | Figla | | Gnaz | | Hmox1 | | Itgb3 | | Ltbp4 | | Mocs1 |
| Acta2 | | Arpc4 | | Cab39l | | Cebpa | | Cpn1 | | Ddah2 | | Ehd1 | | Fkbp11 | | Gnb1 | | Hn1 | | Itgb5 | | Ltbr | | Mocs2 |
| Actl6a | | Arpc5 | | Cacna2d1 | | Cecr5 | | Cpsf2 | | Ddc | | Ehhadh | | Fkbp1b | | Gnb4 | | Hoxa13 | | Itgb7 | | Ltf | | Mpg |
| Actn4 | | Arpp19 | | Cacnb2 | | Celsr1 | | Cpsf3 | | Ddhd1 | | Eif2ak1 | | Fkbp2 | | Gng10 | | Hoxa2 | | Itih2 | | Ltk | | Mpl |
| Actr1a | | Arrb1 | | Cacnb3 | | Cetn3 | | Cpsf4 | | Ddit3 | | Eif2b1 | | Fkbp4 | | Gng11 | | Hoxa5 | | Itm2b | | Luc7l | | Mpp1 |
| Actr1b | | Arsa | | Cacybp | | Cfd | | Cpxm1 | | Ddit4 | | Eif2b2 | | Fkbp7 | | Gng2 | | Hoxa7 | | Ivns1abp | | Lum | | Mpv17 |
| Actr6 | | Art1 | | Calca | | Cfdp1 | | Crabp1 | | Ddost | | Eif2s2 | | Fkbp9 | | Gng4 | | Hoxb3 | | Jag2 | | Lxn | | Mr1 |
| Acy3 | | Art5 | | Calcrl | | Cfl1 | | Creld2 | | Ddr2 | | Eif2s3x | | Fkbpl | | Gnmt | | Hoxb5 | | Jak3 | | Ly6a | | Mras |
| Ada | | Asb3 | | Calm3 | | Cfl2 | | Crem | | Ddt | | Eif4a2 | | Fli1 | | Gnpat | | Hoxb6 | | Jam3 | | Ly6d | | Mrc2 |
| Adam23 | | Asb6 | | Calm4 | | Chad | | Crip2 | | Ddx1 | | Eif4b | | Flot1 | | Golga3 | | Hpcal1 | | Jtb | | Ly86 | | Mrpl11 |
| Adamdec1 | | Asf1a | | Calml4 | | Chaf1a | | Cript | | Ddx21 | | Eif4e | | Flt1 | | Golga5 | | Hpgd | | Kcmf1 | | Ly9 | | Mrpl13 |
| Adamts1 | | Asf1b | | Calr | | Chaf1b | | Crk | | Ddx25 | | Eif4ebp1 | | Flt3l | | Gosr1 | | Hps1 | | Kcnb1 | | Lyl1 | | Mrpl16 |
| Adar | | Asgr2 | | Calu | | Chd1 | | Crkl | | Ddx27 | | Eif4ebp2 | | Flt4 | | Gosr2 | | Hras1 | | Kcnh1 | | Lypla2 | | Mrpl17 |
| Adck1 | | Ash2l | | Camk2d | | Chd1l | | Crtap | | Ddx3x | | Eif4g2 | | Fmnl1 | | Got2 | | Hs1bp3 | | Kcnj2 | | Mad2l1bp | | Mrpl18 |
| Adcy4 | | Asl | | Caml | | Chek2 | | Cryl1 | | Ddx3y | | Elavl1 | | Fmo1 | | Gp5 | | Hsbp1 | | Kcnj4 | | Maea | | Mrpl20 |
| Adcy7 | | Atad1 | | Cant1 | | Cherp | | Crym | | Ddx41 | | Elf1 | | Fmo3 | | Gp9 | | Hsd11b1 | | Kcnj6 | | Maf1 | | Mrpl28 |
| Adcy8 | | Ate1 | | Capn1 | | Chi3l1 | | Cryz | | Ddx47 | | Elf3 | | Fmod | | Gpaa1 | | Hsd11b2 | | Kcnj8 | | Maged1 | | Mrpl3 |
| Adh1 | | Atf2 | | Capn10 | | Chia | | Cs | | Ddx50 | | Elf5 | | Fnbp1 | | Gpc1 | | Hsd17b1 | | Kcnk1 | | Mageh1 | | Mrpl33 |
| Adh7 | | Atf3 | | Capn5 | | Chka | | Cse1l | | Ddx54 | | Ell | | Fnbp4 | | Gpc3 | | Hsd17b12 | | Kcnk2 | | Magoh | | Mrpl34 |
| Adipor1 | | Atm | | Capn6 | | Chmp2a | | Csf1 | | Decr1 | | Eln | | Folr2 | | Gpd1 | | Hsd17b4 | | Kcnk3 | | Mak | | Mrpl35 |
| Adk | | Atp1a1 | | Capn7 | | Chmp7 | | Csf2 | | Dedd | | Elovl5 | | Fos | | Gpd2 | | Hsd17b7 | | Kcnn4 | | Man1a | | Mrpl36 |
| Adm | | Atp1b1 | | Capza1 | | Chordc1 | | Csf2ra | | Dek | | Elovl6 | | Fosl1 | | Gpr172b | | Hspa1l | | Kdelr2 | | Man2a1 | | Mrpl37 |
| Adpgk | | Atp1b3 | | Car3 | | Chpt1 | | Csf2rb2 | | Des | | Elp3 | | Foxa3 | | Gpr65 | | Hspb2 | | Kdelr3 | | Man2b1 | | Mrpl38 |
| Adprh | | Atp2a1 | | Carhsp1 | | Chrac1 | | Csk | | Dffa | | Emb | | Foxf1a | | Gpx2 | | Hspb8 | | Keap1 | | Man2c1 | | Mrpl43 |
| Adra1a | | Atp2a2 | | Cask | | Chrd | | Csnk1d | | Dgat1 | | Emp3 | | Foxf2 | | Gpx3 | | Htatip2 | | Khdrbs1 | | Map1lc3b | | Mrpl49 |

## Slide 2
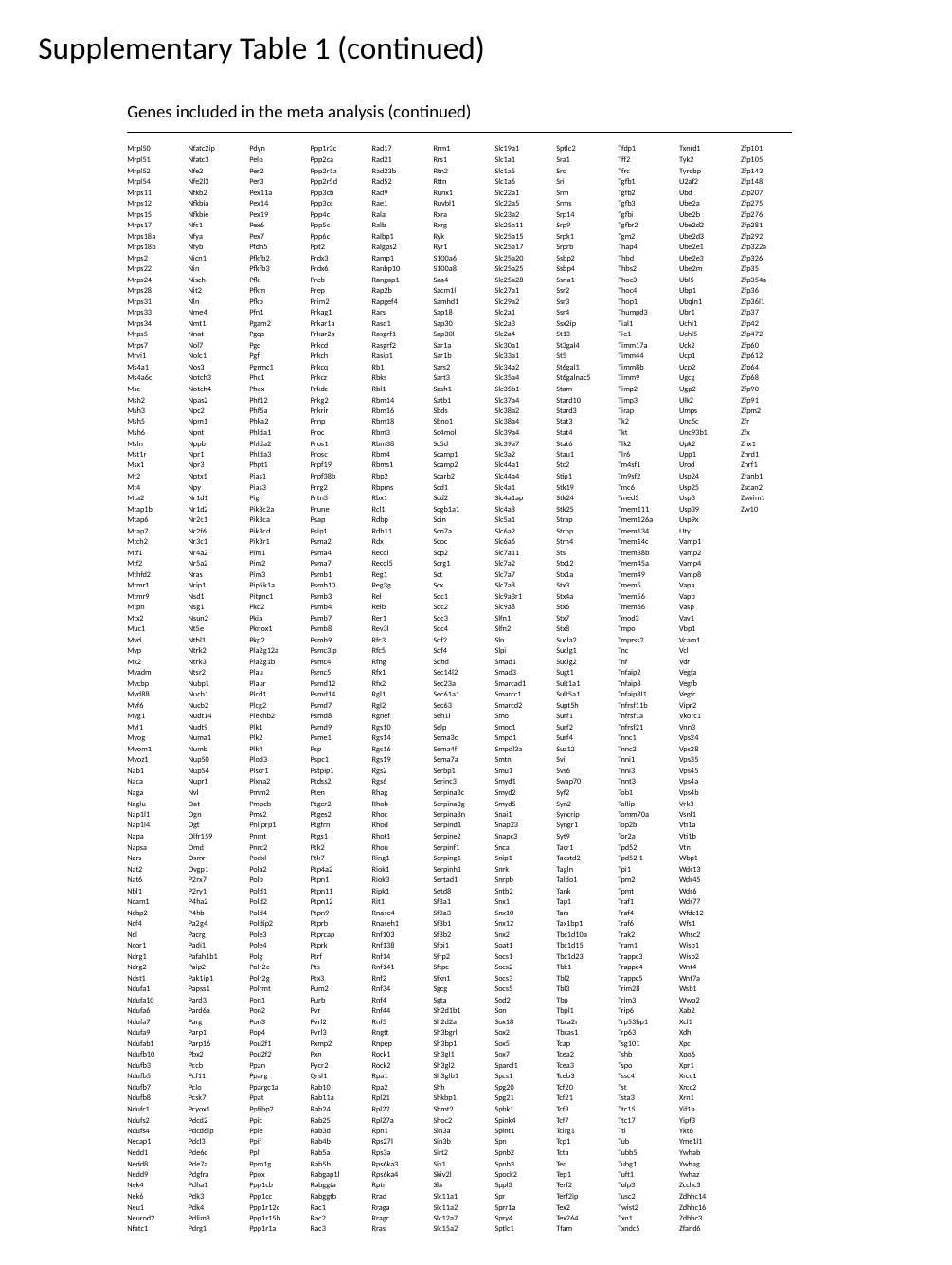

Supplementary Table 1 (continued)
| Genes included in the meta analysis (continued) | | | | | | | | | | | | | | | | | | | | |
| --- | --- | --- | --- | --- | --- | --- | --- | --- | --- | --- | --- | --- | --- | --- | --- | --- | --- | --- | --- | --- |
| | | | | | | | | | | | | | | | | | | | | |
| Mrpl50 | | Nfatc2ip | | Pdyn | | Ppp1r3c | | Rad17 | | Rrm1 | | Slc19a1 | | Sptlc2 | | Tfdp1 | | Txnrd1 | | Zfp101 |
| Mrpl51 | | Nfatc3 | | Pelo | | Ppp2ca | | Rad21 | | Rrs1 | | Slc1a1 | | Sra1 | | Tff2 | | Tyk2 | | Zfp105 |
| Mrpl52 | | Nfe2 | | Per2 | | Ppp2r1a | | Rad23b | | Rtn2 | | Slc1a5 | | Src | | Tfrc | | Tyrobp | | Zfp143 |
| Mrpl54 | | Nfe2l3 | | Per3 | | Ppp2r5d | | Rad52 | | Rttn | | Slc1a6 | | Sri | | Tgfb1 | | U2af2 | | Zfp148 |
| Mrps11 | | Nfkb2 | | Pex11a | | Ppp3cb | | Rad9 | | Runx1 | | Slc22a1 | | Srm | | Tgfb2 | | Ubd | | Zfp207 |
| Mrps12 | | Nfkbia | | Pex14 | | Ppp3cc | | Rae1 | | Ruvbl1 | | Slc22a5 | | Srms | | Tgfb3 | | Ube2a | | Zfp275 |
| Mrps15 | | Nfkbie | | Pex19 | | Ppp4c | | Rala | | Rxra | | Slc23a2 | | Srp14 | | Tgfbi | | Ube2b | | Zfp276 |
| Mrps17 | | Nfs1 | | Pex6 | | Ppp5c | | Ralb | | Rxrg | | Slc25a11 | | Srp9 | | Tgfbr2 | | Ube2d2 | | Zfp281 |
| Mrps18a | | Nfya | | Pex7 | | Ppp6c | | Ralbp1 | | Ryk | | Slc25a15 | | Srpk1 | | Tgm2 | | Ube2d3 | | Zfp292 |
| Mrps18b | | Nfyb | | Pfdn5 | | Ppt2 | | Ralgps2 | | Ryr1 | | Slc25a17 | | Srprb | | Thap4 | | Ube2e1 | | Zfp322a |
| Mrps2 | | Nicn1 | | Pfkfb2 | | Prdx3 | | Ramp1 | | S100a6 | | Slc25a20 | | Ssbp2 | | Thbd | | Ube2e3 | | Zfp326 |
| Mrps22 | | Nin | | Pfkfb3 | | Prdx6 | | Ranbp10 | | S100a8 | | Slc25a25 | | Ssbp4 | | Thbs2 | | Ube2m | | Zfp35 |
| Mrps24 | | Nisch | | Pfkl | | Preb | | Rangap1 | | Saa4 | | Slc25a28 | | Ssna1 | | Thoc3 | | Ubl5 | | Zfp354a |
| Mrps28 | | Nit2 | | Pfkm | | Prep | | Rap2b | | Sacm1l | | Slc27a1 | | Ssr2 | | Thoc4 | | Ubp1 | | Zfp36 |
| Mrps31 | | Nln | | Pfkp | | Prim2 | | Rapgef4 | | Samhd1 | | Slc29a2 | | Ssr3 | | Thop1 | | Ubqln1 | | Zfp36l1 |
| Mrps33 | | Nme4 | | Pfn1 | | Prkag1 | | Rars | | Sap18 | | Slc2a1 | | Ssr4 | | Thumpd3 | | Ubr1 | | Zfp37 |
| Mrps34 | | Nmt1 | | Pgam2 | | Prkar1a | | Rasd1 | | Sap30 | | Slc2a3 | | Ssx2ip | | Tial1 | | Uchl1 | | Zfp42 |
| Mrps5 | | Nnat | | Pgcp | | Prkar2a | | Rasgrf1 | | Sap30l | | Slc2a4 | | St13 | | Tie1 | | Uchl5 | | Zfp472 |
| Mrps7 | | Nol7 | | Pgd | | Prkcd | | Rasgrf2 | | Sar1a | | Slc30a1 | | St3gal4 | | Timm17a | | Uck2 | | Zfp60 |
| Mrvi1 | | Nolc1 | | Pgf | | Prkch | | Rasip1 | | Sar1b | | Slc33a1 | | St5 | | Timm44 | | Ucp1 | | Zfp612 |
| Ms4a1 | | Nos3 | | Pgrmc1 | | Prkcq | | Rb1 | | Sars2 | | Slc34a2 | | St6gal1 | | Timm8b | | Ucp2 | | Zfp64 |
| Ms4a6c | | Notch3 | | Phc1 | | Prkcz | | Rbks | | Sart3 | | Slc35a4 | | St6galnac5 | | Timm9 | | Ugcg | | Zfp68 |
| Msc | | Notch4 | | Phex | | Prkdc | | Rbl1 | | Sash1 | | Slc35b1 | | Stam | | Timp2 | | Ugp2 | | Zfp90 |
| Msh2 | | Npas2 | | Phf12 | | Prkg2 | | Rbm14 | | Satb1 | | Slc37a4 | | Stard10 | | Timp3 | | Ulk2 | | Zfp91 |
| Msh3 | | Npc2 | | Phf5a | | Prkrir | | Rbm16 | | Sbds | | Slc38a2 | | Stard3 | | Tirap | | Umps | | Zfpm2 |
| Msh5 | | Npm1 | | Phka2 | | Prnp | | Rbm18 | | Sbno1 | | Slc38a4 | | Stat3 | | Tk2 | | Unc5c | | Zfr |
| Msh6 | | Npnt | | Phlda1 | | Proc | | Rbm3 | | Sc4mol | | Slc39a4 | | Stat4 | | Tkt | | Unc93b1 | | Zfx |
| Msln | | Nppb | | Phlda2 | | Pros1 | | Rbm38 | | Sc5d | | Slc39a7 | | Stat6 | | Tlk2 | | Upk2 | | Zhx1 |
| Mst1r | | Npr1 | | Phlda3 | | Prosc | | Rbm4 | | Scamp1 | | Slc3a2 | | Stau1 | | Tlr6 | | Upp1 | | Znrd1 |
| Msx1 | | Npr3 | | Phpt1 | | Prpf19 | | Rbms1 | | Scamp2 | | Slc44a1 | | Stc2 | | Tm4sf1 | | Urod | | Znrf1 |
| Mt2 | | Nptx1 | | Pias1 | | Prpf38b | | Rbp2 | | Scarb2 | | Slc44a4 | | Stip1 | | Tm9sf2 | | Usp24 | | Zranb1 |
| Mt4 | | Npy | | Pias3 | | Prrg2 | | Rbpms | | Scd1 | | Slc4a1 | | Stk19 | | Tmc6 | | Usp25 | | Zscan2 |
| Mta2 | | Nr1d1 | | Pigr | | Prtn3 | | Rbx1 | | Scd2 | | Slc4a1ap | | Stk24 | | Tmed3 | | Usp3 | | Zswim1 |
| Mtap1b | | Nr1d2 | | Pik3c2a | | Prune | | Rcl1 | | Scgb1a1 | | Slc4a8 | | Stk25 | | Tmem111 | | Usp39 | | Zw10 |
| Mtap6 | | Nr2c1 | | Pik3ca | | Psap | | Rdbp | | Scin | | Slc5a1 | | Strap | | Tmem126a | | Usp9x | | |
| Mtap7 | | Nr2f6 | | Pik3cd | | Psip1 | | Rdh11 | | Scn7a | | Slc6a2 | | Strbp | | Tmem134 | | Uty | | |
| Mtch2 | | Nr3c1 | | Pik3r1 | | Psma2 | | Rdx | | Scoc | | Slc6a6 | | Strn4 | | Tmem14c | | Vamp1 | | |
| Mtf1 | | Nr4a2 | | Pim1 | | Psma4 | | Recql | | Scp2 | | Slc7a11 | | Sts | | Tmem38b | | Vamp2 | | |
| Mtf2 | | Nr5a2 | | Pim2 | | Psma7 | | Recql5 | | Scrg1 | | Slc7a2 | | Stx12 | | Tmem45a | | Vamp4 | | |
| Mthfd2 | | Nras | | Pim3 | | Psmb1 | | Reg1 | | Sct | | Slc7a7 | | Stx1a | | Tmem49 | | Vamp8 | | |
| Mtmr1 | | Nrip1 | | Pip5k1a | | Psmb10 | | Reg3g | | Scx | | Slc7a8 | | Stx3 | | Tmem5 | | Vapa | | |
| Mtmr9 | | Nsd1 | | Pitpnc1 | | Psmb3 | | Rel | | Sdc1 | | Slc9a3r1 | | Stx4a | | Tmem56 | | Vapb | | |
| Mtpn | | Nsg1 | | Pkd2 | | Psmb4 | | Relb | | Sdc2 | | Slc9a8 | | Stx6 | | Tmem66 | | Vasp | | |
| Mtx2 | | Nsun2 | | Pkia | | Psmb7 | | Rer1 | | Sdc3 | | Slfn1 | | Stx7 | | Tmod3 | | Vav1 | | |
| Muc1 | | Nt5e | | Pknox1 | | Psmb8 | | Rev3l | | Sdc4 | | Slfn2 | | Stx8 | | Tmpo | | Vbp1 | | |
| Mvd | | Nthl1 | | Pkp2 | | Psmb9 | | Rfc3 | | Sdf2 | | Sln | | Sucla2 | | Tmprss2 | | Vcam1 | | |
| Mvp | | Ntrk2 | | Pla2g12a | | Psmc3ip | | Rfc5 | | Sdf4 | | Slpi | | Suclg1 | | Tnc | | Vcl | | |
| Mx2 | | Ntrk3 | | Pla2g1b | | Psmc4 | | Rfng | | Sdhd | | Smad1 | | Suclg2 | | Tnf | | Vdr | | |
| Myadm | | Ntsr2 | | Plau | | Psmc5 | | Rfx1 | | Sec14l2 | | Smad3 | | Sugt1 | | Tnfaip2 | | Vegfa | | |
| Mycbp | | Nubp1 | | Plaur | | Psmd12 | | Rfx2 | | Sec23a | | Smarcad1 | | Sult1a1 | | Tnfaip8 | | Vegfb | | |
| Myd88 | | Nucb1 | | Plcd1 | | Psmd14 | | Rgl1 | | Sec61a1 | | Smarcc1 | | Sult5a1 | | Tnfaip8l1 | | Vegfc | | |
| Myf6 | | Nucb2 | | Plcg2 | | Psmd7 | | Rgl2 | | Sec63 | | Smarcd2 | | Supt5h | | Tnfrsf11b | | Vipr2 | | |
| Myg1 | | Nudt14 | | Plekhb2 | | Psmd8 | | Rgnef | | Seh1l | | Smo | | Surf1 | | Tnfrsf1a | | Vkorc1 | | |
| Myl1 | | Nudt9 | | Plk1 | | Psmd9 | | Rgs10 | | Selp | | Smoc1 | | Surf2 | | Tnfrsf21 | | Vnn3 | | |
| Myog | | Numa1 | | Plk2 | | Psme1 | | Rgs14 | | Sema3c | | Smpd1 | | Surf4 | | Tnnc1 | | Vps24 | | |
| Myom1 | | Numb | | Plk4 | | Psp | | Rgs16 | | Sema4f | | Smpdl3a | | Suz12 | | Tnnc2 | | Vps28 | | |
| Myoz1 | | Nup50 | | Plod3 | | Pspc1 | | Rgs19 | | Sema7a | | Smtn | | Svil | | Tnni1 | | Vps35 | | |
| Nab1 | | Nup54 | | Plscr1 | | Pstpip1 | | Rgs2 | | Serbp1 | | Smu1 | | Svs6 | | Tnni3 | | Vps45 | | |
| Naca | | Nupr1 | | Plxna2 | | Ptdss2 | | Rgs6 | | Serinc3 | | Smyd1 | | Swap70 | | Tnnt3 | | Vps4a | | |
| Naga | | Nvl | | Pmm2 | | Pten | | Rhag | | Serpina3c | | Smyd2 | | Syf2 | | Tob1 | | Vps4b | | |
| Naglu | | Oat | | Pmpcb | | Ptger2 | | Rhob | | Serpina3g | | Smyd5 | | Syn2 | | Tollip | | Vrk3 | | |
| Nap1l1 | | Ogn | | Pms2 | | Ptges2 | | Rhoc | | Serpina3n | | Snai1 | | Syncrip | | Tomm70a | | Vsnl1 | | |
| Nap1l4 | | Ogt | | Pnliprp1 | | Ptgfrn | | Rhod | | Serpind1 | | Snap23 | | Syngr1 | | Top2b | | Vti1a | | |
| Napa | | Olfr159 | | Pnmt | | Ptgs1 | | Rhot1 | | Serpine2 | | Snapc3 | | Syt9 | | Tor2a | | Vti1b | | |
| Napsa | | Omd | | Pnrc2 | | Ptk2 | | Rhou | | Serpinf1 | | Snca | | Tacr1 | | Tpd52 | | Vtn | | |
| Nars | | Osmr | | Podxl | | Ptk7 | | Ring1 | | Serping1 | | Snip1 | | Tacstd2 | | Tpd52l1 | | Wbp1 | | |
| Nat2 | | Ovgp1 | | Pola2 | | Ptp4a2 | | Riok1 | | Serpinh1 | | Snrk | | Tagln | | Tpi1 | | Wdr13 | | |
| Nat6 | | P2rx7 | | Polb | | Ptpn1 | | Riok3 | | Sertad1 | | Snrpb | | Taldo1 | | Tpm2 | | Wdr45 | | |
| Nbl1 | | P2ry1 | | Pold1 | | Ptpn11 | | Ripk1 | | Setd8 | | Sntb2 | | Tank | | Tpmt | | Wdr6 | | |
| Ncam1 | | P4ha2 | | Pold2 | | Ptpn12 | | Rit1 | | Sf3a1 | | Snx1 | | Tap1 | | Traf1 | | Wdr77 | | |
| Ncbp2 | | P4hb | | Pold4 | | Ptpn9 | | Rnase4 | | Sf3a3 | | Snx10 | | Tars | | Traf4 | | Wfdc12 | | |
| Ncf4 | | Pa2g4 | | Poldip2 | | Ptprb | | Rnaseh1 | | Sf3b1 | | Snx12 | | Tax1bp1 | | Traf6 | | Wfs1 | | |
| Ncl | | Pacrg | | Pole3 | | Ptprcap | | Rnf103 | | Sf3b2 | | Snx2 | | Tbc1d10a | | Trak2 | | Whsc2 | | |
| Ncor1 | | Padi1 | | Pole4 | | Ptprk | | Rnf138 | | Sfpi1 | | Soat1 | | Tbc1d15 | | Tram1 | | Wisp1 | | |
| Ndrg1 | | Pafah1b1 | | Polg | | Ptrf | | Rnf14 | | Sfrp2 | | Socs1 | | Tbc1d23 | | Trappc3 | | Wisp2 | | |
| Ndrg2 | | Paip2 | | Polr2e | | Pts | | Rnf141 | | Sftpc | | Socs2 | | Tbk1 | | Trappc4 | | Wnt4 | | |
| Ndst1 | | Pak1ip1 | | Polr2g | | Ptx3 | | Rnf2 | | Sfxn1 | | Socs3 | | Tbl2 | | Trappc5 | | Wnt7a | | |
| Ndufa1 | | Papss1 | | Polrmt | | Pum2 | | Rnf34 | | Sgcg | | Socs5 | | Tbl3 | | Trim28 | | Wsb1 | | |
| Ndufa10 | | Pard3 | | Pon1 | | Purb | | Rnf4 | | Sgta | | Sod2 | | Tbp | | Trim3 | | Wwp2 | | |
| Ndufa6 | | Pard6a | | Pon2 | | Pvr | | Rnf44 | | Sh2d1b1 | | Son | | Tbpl1 | | Trip6 | | Xab2 | | |
| Ndufa7 | | Parg | | Pon3 | | Pvrl2 | | Rnf5 | | Sh2d2a | | Sox18 | | Tbxa2r | | Trp53bp1 | | Xcl1 | | |
| Ndufa9 | | Parp1 | | Pop4 | | Pvrl3 | | Rngtt | | Sh3bgrl | | Sox2 | | Tbxas1 | | Trp63 | | Xdh | | |
| Ndufab1 | | Parp16 | | Pou2f1 | | Pxmp2 | | Rnpep | | Sh3bp1 | | Sox5 | | Tcap | | Tsg101 | | Xpc | | |
| Ndufb10 | | Pbx2 | | Pou2f2 | | Pxn | | Rock1 | | Sh3gl1 | | Sox7 | | Tcea2 | | Tshb | | Xpo6 | | |
| Ndufb3 | | Pccb | | Ppan | | Pycr2 | | Rock2 | | Sh3gl2 | | Sparcl1 | | Tcea3 | | Tspo | | Xpr1 | | |
| Ndufb5 | | Pcf11 | | Pparg | | Qrsl1 | | Rpa1 | | Sh3glb1 | | Spcs1 | | Tceb3 | | Tssc4 | | Xrcc1 | | |
| Ndufb7 | | Pclo | | Ppargc1a | | Rab10 | | Rpa2 | | Shh | | Spg20 | | Tcf20 | | Tst | | Xrcc2 | | |
| Ndufb8 | | Pcsk7 | | Ppat | | Rab11a | | Rpl21 | | Shkbp1 | | Spg21 | | Tcf21 | | Tsta3 | | Xrn1 | | |
| Ndufc1 | | Pcyox1 | | Ppfibp2 | | Rab24 | | Rpl22 | | Shmt2 | | Sphk1 | | Tcf3 | | Ttc15 | | Yif1a | | |
| Ndufs2 | | Pdcd2 | | Ppic | | Rab25 | | Rpl27a | | Shoc2 | | Spink4 | | Tcf7 | | Ttc17 | | Yipf3 | | |
| Ndufs4 | | Pdcd6ip | | Ppie | | Rab3d | | Rpn1 | | Sin3a | | Spint1 | | Tcirg1 | | Ttl | | Ykt6 | | |
| Necap1 | | Pdcl3 | | Ppif | | Rab4b | | Rps27l | | Sin3b | | Spn | | Tcp1 | | Tub | | Yme1l1 | | |
| Nedd1 | | Pde6d | | Ppl | | Rab5a | | Rps3a | | Sirt2 | | Spnb2 | | Tcta | | Tubb5 | | Ywhab | | |
| Nedd8 | | Pde7a | | Ppm1g | | Rab5b | | Rps6ka3 | | Six1 | | Spnb3 | | Tec | | Tubg1 | | Ywhag | | |
| Nedd9 | | Pdgfra | | Ppox | | Rabgap1l | | Rps6ka4 | | Skiv2l | | Spock2 | | Tep1 | | Tuft1 | | Ywhaz | | |
| Nek4 | | Pdha1 | | Ppp1cb | | Rabggta | | Rptn | | Sla | | Sppl3 | | Terf2 | | Tulp3 | | Zcchc3 | | |
| Nek6 | | Pdk3 | | Ppp1cc | | Rabggtb | | Rrad | | Slc11a1 | | Spr | | Terf2ip | | Tusc2 | | Zdhhc14 | | |
| Neu1 | | Pdk4 | | Ppp1r12c | | Rac1 | | Rraga | | Slc11a2 | | Sprr1a | | Tex2 | | Twist2 | | Zdhhc16 | | |
| Neurod2 | | Pdlim3 | | Ppp1r15b | | Rac2 | | Rragc | | Slc12a7 | | Spry4 | | Tex264 | | Txn1 | | Zdhhc3 | | |
| Nfatc1 | | Pdrg1 | | Ppp1r1a | | Rac3 | | Rras | | Slc15a2 | | Sptlc1 | | Tfam | | Txndc5 | | Zfand6 | | |
